# Formation of functional, non‐amyloidogenic fibres by recombinant *Bacillus subtilis* TasA

**DOI:** 10.1111/mmi.13985

**Published:** 2018-11-16

**Authors:** Elliot Erskine, Ryan J. Morris, Marieke Schor, Chris Earl, Rachel M. C. Gillespie, Keith M. Bromley, Tetyana Sukhodub, Lauren Clark, Paul K. Fyfe, Louise C. Serpell, Nicola R. Stanley‐Wall, Cait E. MacPhee

**Affiliations:** ^1^ Division of Molecular Microbiology, School of Life Sciences University of Dundee Dundee DD1 4HN UK; ^2^ James Clerk Maxwell Building, School of Physics, and Astronomy University of Edinburgh, The Kings Buildings, Peter Guthrie Tait Road Edinburgh EH9 3FD UK; ^3^ School of Life Sciences University of Sussex Falmer BN1 9QG UK

## Abstract

Bacterial biofilms are communities of microbial cells encased within a self‐produced polymeric matrix. In the *Bacillus subtilis* biofilm matrix, the extracellular fibres of TasA are essential. Here, a recombinant expression system allows interrogation of TasA, revealing that monomeric and fibre forms of TasA have identical secondary structure, suggesting that fibrous TasA is a linear assembly of globular units. Recombinant TasA fibres form spontaneously, and share the biological activity of TasA fibres extracted from *B. subtilis*, whereas a TasA variant restricted to a monomeric form is inactive and subjected to extracellular proteolysis. The biophysical properties of both native and recombinant TasA fibres indicate that they are not functional amyloid‐like fibres. A gel formed by TasA fibres can recover after physical shear force, suggesting that the biofilm matrix is not static and that these properties may enable *B. subtilis* to remodel its local environment in response to external cues. Using recombinant fibres formed by TasA orthologues we uncover species variability in the ability of heterologous fibres to cross‐complement the *B. subtilis tasA* deletion. These findings are indicative of specificity in the biophysical requirements of the TasA fibres across different species and/or reflect the precise molecular interactions needed for biofilm matrix assembly.

## Introduction

Biofilms are communities of microbial cells that underpin diverse processes including sewage bioremediation, plant growth promotion, chronic infections and industrial biofouling (Costerton *et al*., 1987). The microbial cells resident in the biofilm are encased within a self‐produced extracellular polymeric matrix that commonly comprises lipids, proteins, extracellular DNA and exopolysaccharides (Flemming and Wingender, 2010; Hobley *et al*., 2015). This matrix fulfils a variety of functions for the community, from providing structural rigidity and protection from the external environment, to supporting signal transduction and nutrient adsorption (Flemming and Wingender, 2010; Dragoš and Kovács, 2017; Vidakovic *et al*., 2018). *Bacillus subtilis* is a soil dwelling bacterium that is a model for biofilm formation by Gram‐positive bacteria; beyond this it is of commercial interest due to its biocontrol and plant growth promoting properties that highlight its potential to substitute for petrochemical derived pesticides and fertilizers (Bais *et al*., 2004; Chen *et al*., 2012, 2013). Biofilm formation is subject to complex regulatory pathways (Cairns *et al*., 2014) and it is known that the *B. subtilis* biofilm matrix predominantly comprises three specific components. The first is an exopolysaccharide that serves to retain moisture within the biofilm and functions as a signalling molecule (Seminara *et al*., 2012; Elsholz *et al*., 2014). The composition of the exopolysaccharide remains unclear due to three inconsistent monosaccharide composition analyses being detailed thus far (Chai *et al*., 2012; Jones *et al*., 2014; Roux *et al*., 2015). The second component is the protein BslA that is responsible for the non‐wetting nature of the biofilm (Kobayashi and Iwano 2012; Hobley *et al*., 2013; Bromley *et al*., 2015) and for biofilm architecture, independently of its ability to render the surface of the biofilm water‐repellent (Arnaouteli *et al*., 2017). The third component of the biofilm matrix is the protein TasA (together with accessory protein TapA) that is needed for biofilm structure including attachment to plant roots (Branda *et al*., 2004; Romero *et al*., 2011; Beauregard *et al*., 2013).

TasA is a product of the *tapA‐sipW‐tasA* locus (Michna et al., 2016). It is post‐translationally modified by SipW (Stöver and Driks, 1999), a specialized signal peptidase that releases the mature 261‐amino acid TasA into the extracellular environment where it forms long protein fibres that contribute to the superstructure of the biofilm matrix and are needed for biofilm integrity (Branda *et al*., 2006; Romero *et al*., 2010). In addition to functions involved in the process of biofilm formation, TasA is also linked with sliding motility (van Gestel *et al*., 2015) and spore coat formation (Stöver and Driks, 1999; Serrano *et al*., 1999). TasA fibres can be extracted from *B. subtilis* biofilms, and exogenous provision to a *tasA* null strain has previously been reported to reinstate structure to floating pellicles (Romero *et al*., 2010). Due to the reported ability of TasA fibres to bind the dyes Congo Red and Thioflavin T (ThT), *ex vivo* purified TasA fibres have previously been classified as functional bacterial amyloid fibres (Romero *et al*., 2010), placing them alongside the curli fibres of *E. coli* (Chapman *et al*., 2002).

Amyloid‐like fibres are well‐known for their association with diseases like Alzheimer's and Parkinson's (Eisenberg and Jucker, 2012). In these conditions, highly stable fibrillar protein deposits are found in tissue sections, and are associated with cell damage (Hardy and Selkoe, 2002). The amyloid fibres in these deposits are characterised by several properties: (i) β‐sheet‐rich structures that are assembled into the canonical ‘cross‐β’ structure; (ii) the ability of the fibres to bind the dye Congo Red and exhibit green birefringence under cross polarised light; (iii) kinetics of formation that indicate a nucleated self‐assembly process and (iv) a fibril structure that is unbranched, 6–12nm in diameter, and often microns in length (Sunde and Blake, 1997; Sipe *et al*., 2016). Once formed, these protein aggregates are highly stable, and in many cases are thought to be the lowest energy structural form shorter polypeptide chains can adopt (Baldwin *et al*., 2011). ‘Functional’ amyloid fibres refer to structurally robust, protease and SDS resistant fibrillar protein deposits that share the characteristic structural properties of amyloid fibres, but are beneficial to the organism rather than being associated with disease (Fowler *et al*., 2007). Significant caution is required in identifying functional amyloid‐like fibres from predominantly *in vitro* data however, as many proteins and peptides can be induced to adopt the canonical amyloid fibre cross‐β fold through appropriate manipulation of solution conditions such as changes in pH, temperature, cosolvent, salt or the presence of an interface (Kayed *et al*., 1999; Ferrão‐Gonzales *et al*., 2000; Uversky *et al*., 2001; Hong *et al*., 2006; Kalapothakis *et al*., 2015), which may or may not be of physiological relevance. Indeed, the ability of proteins to assemble into the cross‐β architecture appears to be a ‘generic’ property of the polypeptide chain, independent of the amino acid sequence or the native structure of the precursor (Dobson, 1999; MacPhee and Dobson, 2000).

Here, we show that, although TasA is a fibre‐forming protein, it is not amyloid‐like in character. We have produced recombinant TasA in both fibre and monomeric forms, and show that the secondary structures of these are both identical to each other and to those reported previously for the exogenous purified TasA fibres (Romero *et al*., 2010; Chai *et al*., 2013), appearing significantly helical in character. We have also examined native TasA fibres in enriched extracts from *B. subtilis* and show that both the native and recombinant forms of fibrous TasA show indistinguishable biological activity, being able to reinstate biofilm structure to a Δ*tasA sinR* deletion strain. X‐ray fibre diffraction of the recombinant TasA fibres shows that they are assembled from a helical repeat of globular protein units arranged approximately 45 Å apart, and the data are not consistent with the canonical ‘cross‐β’ diffraction pattern associated with amyloid‐like fibres. Neither monomeric nor fibrous forms of recombinant TasA bind the dyes Congo Red or ThT, and although TasA‐enriched extracts from *B. subtilis* biofilms show both Congo Red and ThT binding activity, this is at a similar level to that produced by protein extracts from cells lacking *tasA*. Thus, TasA does not fall into the class of ‘functional amyloid‐like fibres’; nonetheless it plays a critical role in biofilm structure.

## Results

### TasA forms non‐amyloid fibres and is rendered monomeric by placement of a single N‐terminal amino acid

**Figure 1:**
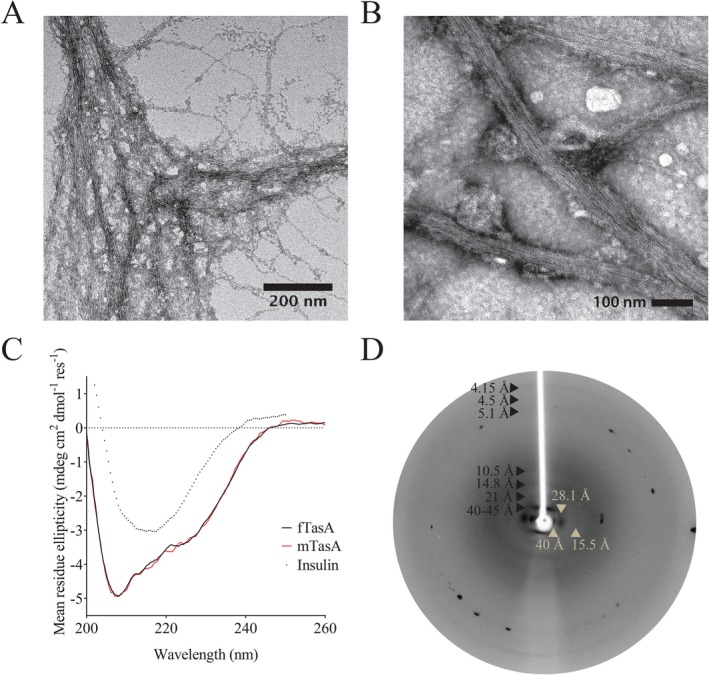
Recombinant TasA forms fibres. A and B. Transmission electron microscopy images of recombinant fTasA and nTasA(+) stained with uranyl acetate shows the presence of fibres several microns in length and approximately 15nm wide. C. Solution state circular dichroism spectra of recombinant fTasA (Solid black line) and mTasA (Solid red line). Spectrum of insulin in the amyloid fibre form is shown for comparison (Dotted black line). D. X‐Ray Diffraction of recombinant fTasA protein fibres with exposure for 60s where meridional and equatorial diffraction signals are indicated in black and beige respectively.

We predicted the identity of the N‐terminus of mature TasA protein *in silico* using SignalP v4.2 (Petersen *et al*., 2011) and subsequently confirmed this *in vivo* using mass spectrometry. Based on this information we designed an expression construct to allow purification of recombinant *B. subtilis* TasA (Supporting Information Fig. S1A), corresponding to the mature TasA sequence covering amino acids 28–261, after production in *E. coli* (Supporting Information Fig. S1B). The purified protein displayed obvious viscosity, not flowing upon inversion of the tube, and bead tracking microrheology confirmed the gel‐like nature of the solution (Supporting Information Fig. S1C). This viscosity arises from the formation of a fibrous aggregate that can be characterised by transmission electron microscopy (TEM) (Fig. 1A). Within these fibres we observed a subunit repeat along the fibre axis, repeating at approximately 4nm. Hereafter we refer to this protein as ‘fTasA’ for fibrous TasA. To compare the recombinant protein to the native form, we extracted TasA fibres from *B. subtilis* strain NRS5422 which we refer to as native extract TasA positive ‘nTasA(+)’. To identify the specific contribution of TasA originating from this partially purified, heterogeneous sample we followed the same enrichment process with a strain carrying a *tasA* deletion (strain NRS5931, this sample is designated the native extract TasA negative sample, hereafter ‘nTasA(−)’ (Supporting Information Fig. S1D and E). The subunit repeat pattern seen in the recombinant TasA fibres was also visible in the nTasA(+) fibres (Fig 1B) and no comparable fibres were observed in the nTasA(–) sample (Supporting Information Fig. S1F).

Circular dichroism (CD) spectroscopy of fTasA shows a minimum at 208nm, a shoulder at ∼222nm, and a maximum below 200nm (Fig. 1C). The overall shape and the position of the minima are consistent with a predominantly (>50%) α‐helical conformation, however the ratio of the two minima suggests there are likely to be contributions from other secondary structural elements. The spectrum resembles that previously reported for TasA oligomers (Chai *et al*., 2013) or fibres purified directly from *B. subtilis* (Romero *et al*., 2010), and does not display the high β‐sheet content (represented by a single minimum at ∼216–218nm) typical for proteins in amyloid‐like fibres; nor do the measured minima/maximum correspond to those predicted or measured for highly twisted β‐sheets (Micsonai *et al*., 2015). It was not possible to obtain a CD spectrum for the natively extracted nTasA(+) due to contamination with flagella, which were also visible in TEM images (Supporting Information Fig. S1F, arrow), and identified due to their uniformity in width, lack of branching in the filaments and their presence in the nTasA(−) extract. Other proteins were also identified by mass spectrometry as detailed in the legend relating to Supporting Information Fig. S1D.

X‐ray fibre diffraction from partially aligned fTasA fibres (Fig. 1D) showed a series of layer lines on the meridian. The lowest resolution visible was 41–45 Å/4.1–4.5nm and further strong layer lines were measured at 21 Å and 14.8 Å. A weak, high resolution meridional diffraction signal was observed at 4.15 Å. These spacings are consistent with a helical or globular repeat distance of 40–45 Å, as was observed by TEM for both fTasA and nTasA(+). On the equator of the pattern, strong diffraction signals were measured at approx. 40 Å, 28.1 Å and 15.5 Å. These spacings are consistent with packing of a fibre with globular units of dimensions 28 × 45 Å. The diffraction signals expected for a cross‐ β amyloid‐like structure [i.e., 4.7 Å (the inter‐strand distance) and ∼10–12 Å (the inter‐sheet distance) (O Sumner Makin and Serpell, 2005)] were not observed.

Many amyloid‐like fibres bind the dyes Congo Red and ThT and dye‐binding assays are often used to assess fibril assembly, so we next tested whether our recombinant protein fTasA and our *B. subtilis* nTasA(+) extract bound these dyes. ThT fluorescence in the presence of fTasA was similar to that of a non‐fibrillar control protein (Supporting Information Fig. S1G), and showed no evidence of an interaction with Congo Red (Supporting Information Fig. S1H; fibrils assembled from insulin are shown as a positive control). The nTasA(+) extract enhanced ThT fluorescence and showed Congo Red binding (Supporting Information Fig. S1G and H), however so did the nTasA(−) extract from the strain carrying the *tasA* deletion (Supporting Information Fig. S1G and H). Therefore, there is no evidence that fibre‐forming TasA binds ThT or Congo Red, whether recombinant or extracted from *B. subtilis*. Thus, the combination of the visible subunit repeat in both recombinant and native TasA fibres, the absence of a clear β‐sheet secondary structure in the recombinant protein (as has also been reported for *ex vivo* TasA), susceptibility to SDS treatment, the X‐ray fibre diffraction results, and the lack of dye binding, all suggest that TasA is not a functional amyloid fibre (see *Discussion*).

Through addition of a single amino acid to the N‐terminus of the mature TasA protein (Supporting Information Fig. S2A), we discovered that it was possible to block fibre formation *in vitro*. Inhibition of TasA fibre assembly was not dependent on the chemical properties of the added amino acid, with lysine, phenylalanine, glutamic acid, alanine, or serine all being effective (Supporting Information Fig. S2B and S2D). Having compared the behaviour of these proteins at this level we focussed our subsequent analysis on the purified monomeric serine‐tagged TasA (Supporting Information Fig. S2C). Size‐exclusion chromatography (SEC) showed a single peak of around 30 kDa (Supporting Information Fig. S2D), and the molecular mass was confirmed by LC‐MS, ensuring the specificity of the protein sequence. Monomeric TasA (‘mTasA’) was not viscous, with bead tracking microrheology confirming the liquid‐like nature of the purified protein solution (Supporting Information Fig. S1C); moreover, no fibres were apparent by TEM. The monomeric protein showed the same lack of ThT binding as fTasA (Supporting Information Fig. S1G). The CD spectrum of mTasA was indistinguishable from fTasA (Fig. 1C) indicating that addition of a single amino acid to the N‐terminus did not affect the secondary structure, and moreover suggesting that the fibrous form is likely constructed from a linear assembly of these monomeric units. Thus, examination of TasA form and function in both fibrous and monomeric states was possible.

### Recombinant TasA fibres are biologically active

**Figure 2:**
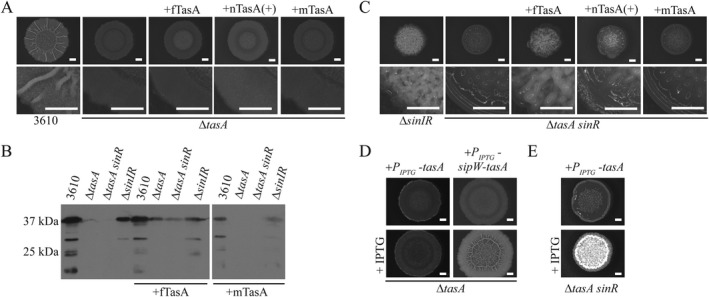
Recombinant fTasA is biologically active. A, C. Biofilm phenotypes of wild type (NCIB3610), Δ*tasA* (NRS5267), Δ*sinIR* (NRS2012) and Δ*tasA sinR* (NRS5248) strains with the addition of 10 µg fTasA, 30 µg nTasA(+) or 10 µg mTasA as indicated. B. Immunoblot blot analysis of biofilm lysate collected from biofilms challenged with α‐TasA antibody. D, E. Biofilm phenotype of ΔtasA and Δ*tasA sinR* complementation in presence of 100 µM for Δ*tasA* P_IPTG_‐*tasA* (NRS5276) and 25 µM for Δ*tasA sinR* P_IPTG_‐*tasA* (NRS5255) and Δ*tasA* P_IPTG_‐*sipW‐tasA* (NRS5313). The scale bar represents 2 mm.

To test the biological functionality of the recombinant TasA fibres, an in frame Δ*tasA* deletion (NRS5267) was constructed. Exogenous addition of neither recombinant fTasA (Supporting Information Fig. S3A), nor purified nTasA(+) successfully reinstated biofilm architecture to the Δ*tasA* mutant (Fig. 2A, Supporting Information Fig. S3B). Immunoblot analysis of whole biofilm protein extracts using anti‐TasA antibodies revealed that while fTasA and nTasA(+) do not influence biofilm morphology, when cultured with the *tasA* deletion strain, the exogenously added proteins are still detectable after 48h incubation (Fig. 2B, Supporting Information Fig S3C). The biofilm phenotype was similarly unchanged when monomeric TasA or nTasA(−) was exogenously added to the Δ*tasA* strain (Fig. 2A, Supporting Information Fig S3B). For the monomeric protein, we considered two possibilities: (i) mTasA is unstable in the presence of cells when added exogenously or (ii) mTasA is stable, but not functional, suggesting that it is not converted into a functional form following exogenous addition to the biofilm. Immunoblot analysis of protein extracts using anti‐TasA antibodies revealed that exogenously added mTasA reached undetectable levels after incubation with the *tasA* strain during biofilm formation conditions (Fig. 2B). These findings indicate that mTasA is likely to be degraded by proteolysis.

*Bacillus subtilis* secretes 7 heat‐labile proteases into the extracellular environment (Sloma *et al*., 1988; Rufo *et al*., 1990; Sloma *et al*., 1990, 1991; Wu *et al*., 1990; Tran *et al*., 1991; Margot and Karamata, 1996) to which mTasA could be exposed. Therefore we analysed the stability of recombinant fTasA and mTasA after incubation in cell‐free spent culture supernatant derived from planktonic growth of NCIB3610 to stationary phase. This revealed that assembly of TasA into the fibre form confers protection from degradation (Supporting Information Fig. S3D and E). We then assessed stability of mTasA and fTasA protein in spent culture supernatant isolated from a laboratory prototrophic strain (PY79) and two derivatives of PY79 that lacked the coding regions for secreted extracellular proteases: namely strains in which the genes for six (‘Δ6’) or seven (‘Δ7’) of the native proteases had been deleted (Supporting Information Table S1). While fTasA was detected under all incubation conditions (Supporting Information Fig. S3D), mTasA was only observed when the culture supernatant had been heat treated to denature the protein content or when it was incubated with the spent culture supernatant derived from Δ6 and Δ7 exoprotease‐deficient strains (Supporting Information Fig. S3E). These results indicate susceptibility of the monomeric TasA protein to proteolysis and that protection is conferred by self‐assembly to a fibre form.

SinR is a major repressor of biofilm formation that functions, in part, by directly inhibiting transcription from the operons needed for the production of the exopolysaccharide and TasA fibres, both essential components of the *B. subtilis* matrix (Chu *et al*., 2006). Deletion of *sinR* results in a biofilm that is densely wrinkled and highly adherent to a surface when compared to the parental strain, due to increased production of the biofilm macromolecules (Fig. 2C, Supporting Information Fig. S3B). A Δ*tasA sinR* deletion strain displays a flat, featureless biofilm by comparison with a *sinR* deletion strain (Fig. 2C, Supporting Information Fig. S3B). We found that addition of 10 µg recombinant fTasA or 30 µg of nTasA(+) extract broadly returned the wrinkled *sinR* mutant‐like phenotype to the Δ*tasA sinR* mutant (Fig. 2C, Supporting Information Fig. S3B). Thus, the recombinant form of fTasA is biologically functional, and shows the same functional activity as native TasA. In contrast, monomeric TasA (Supporting Information Fig. S2A) and the nTasA(−) samples did not reinstate rugosity to the Δ*tasA sinR* deletion strain (Fig. 2C, Supporting Information Fig. S3B) suggesting that *in vivo* templating of mTasA into a functional fibrous form does not occur and that the activity in the nTasA(+) sample was linked to TasA activity specifically. As was observed when protein was supplied exogenously to the single *tasA* deletion strain, mTasA was not detectable by anti‐TasA immunoblot analysis after co‐incubation with the Δ*tasA sinR* deletion strain, but fTasA was detected (Fig. 2B), confirming the susceptibility of mTasA to proteolysis.

We cannot explain why fTasA and nTasA(+) do not recover biofilm rugosity to the *tasA* deletion when supplied exogenously. However, we note that the *tasA* and *tasA sinR* strains differ in the requirements needed for genetic complementation. The *tasA* deletion cannot be genetically complemented by expression of *tasA* under the control of an inducible promoter at the ectopic *amyE* locus (Fig 2D, Supporting Information Fig. S3F) but requires co‐expression of *sipW* and *tasA* to return biofilm formation to a wild‐type morphology (Fig 2D, Supporting Information Fig. S3F). This is not an indication that *sipW* is inadvertently disrupted in the *tasA* strain, as restoration of biofilm formation by the *tasA* mutant was equally successful using a complementation construct when codons 3 and 4 of *sipW* were replaced with stop codons (Supporting Information Fig. S3F). In contrast, provision of the *tasA* coding region only at the ectopic *amyE* locus in the Δ*tasA sinR* deletion strain (NRS5255) is sufficient to allow a densely wrinkled biofilm structure to be recovered (Fig 2E, Supporting Information Fig. S3G). We next explored the mechanism underpinning the interaction between fibrous TasA and the components of the biofilm.

### Recombinant TasA fibres require the biofilm exopolysaccharide for activity

**Figure 3:**
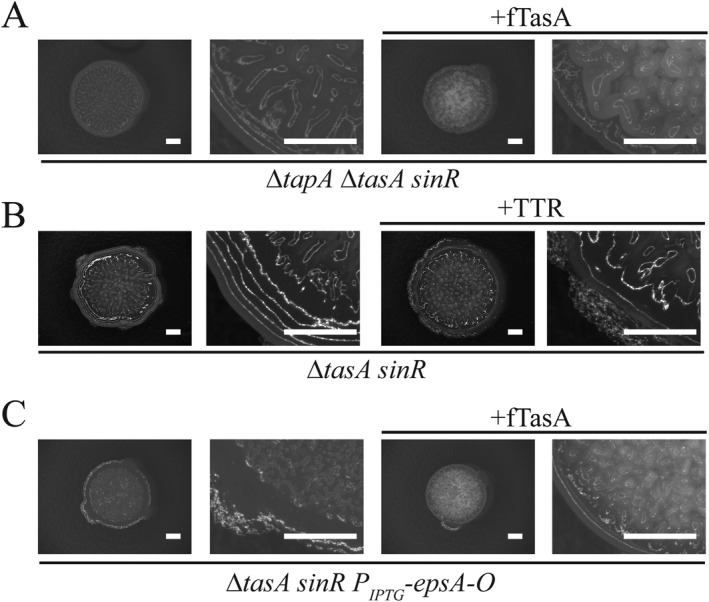
Recombinant fTasA does not need TapA but required the biofilm exopolysaccharide for activity. A. Biofilm phenotype of Δ*tapA* Δ*tasA sinR* (NRS5749) mutant upon addition of 10 µg fTasA *ex vivo*. B. Biofilm phenotype of Δ*tasA sinR* (NRS5248) upon addition of transthyretin (TTR). C. Biofilm phenotype of Δ*tasA sinR* P_IPTG_‐*epsA‐O* (NRS5421) strain in the presence of 100 µM IPTG in absence and presence of *ex vivo* addition of 10 µg fTasA. An enlarged section of bottom left corner of the biofilm is shown in each case. The scale bar represents 2 mm.

TasA protein fibres have been reported to be anchored to the cell wall *via* an interaction with a partner protein called TapA (Romero *et al*., 2011). Moreover, deletion of *tapA* is associated with a reduction in the level of TasA (Romero *et al*., 2014). As the deletion of *sinR* leads to increased transcription of the entire *tapA* operon (Chu *et al*., 2006), we hypothesised that there may be an increase in available TapA ‘docking’ sites available for the anchoring of TasA fibres when added *ex vivo* to the Δ*tasA sinR* double mutant. To test if TapA is needed for wrinkling of the Δ*tasA sinR* deletion strain upon addition of preassembled TasA fibres, we constructed a Δ*tapA* Δ*tasA sinR* triple deletion strain (Fig. 3A, Supporting Information Fig. S4A). This strain could be returned to the *sinR* morphology upon genetic complementation with the *tapA‐sipW‐tasA* gene cluster at an ectopic location in the chromosome (Supporting Information Fig. S4B). When fTasA was co‐cultured with the Δ*tapA* Δ*tasA sinR* strain, we observed similar levels of *ex vivo* complementation as when fTasA was added to the Δ*tasA sinR* deletion strain (compare Figs 2C and 3A), thus suggesting that TapA is not required to reinstate biofilm architecture when fully formed TasA fibres are supplied.

In light of the findings above, we explored if the rugosity displayed by the Δ*tasA sinR* in the presence of *ex vivo* recombinant fTasA was due to a specific interaction with the matrix components, or whether the presence of sufficient fibrous material is enough to confer rugosity simply due to the gelatinous nature of the concentrated fTasA protein. To test this, we took two approaches. First we tested if an entirely unrelated protein fibre could substitute for fTasA, simply by provision of a fibrous protein scaffold. We provided amyloid‐like fibres assembled from the well‐characterised transthyretin peptide 105–115 (TTR_105–115_) (Fitzpatrick *et al*., 2013) exogenously to the Δ*tasA sinR* strain followed by incubation under biofilm forming conditions. Despite the obvious viscosity of the TTR_105–115_ gel, it did not reinstate biofilm rugosity (Fig. 3B, Supporting Information Fig. S4D). Therefore, a biochemically distinct fibre cannot substitute for fTasA. Next, we assessed whether the biofilm exopolysaccharide was needed for rugosity under these conditions. This experiment was based on the premise that if the wrinkle formation after addition of exogenous fTasA was derived from the gelling properties of fTasA, the exopolysaccharide would not be needed. To determine this we used a strain where the entire *eps*A‐O operon was placed under the control of an IPTG inducible promoter at the native location on the chromosome (Terra *et al*., 2012). We then added fTasA with and without induction of the *epsA‐O* operon. Analysis of the biofilm phenotypes revealed that we were able to induce rugosity with fTasA only in the presence of IPTG (Fig. 3C, Supporting Information Fig. S4E), although not to the same extent as seen in the parent strain – most likely because production of the exopolysaccharide is uncoupled from its native regulation circuitry, impacting the level of polymer produced. Therefore we can conclude that both the biofilm exopolysaccharide and TasA are required to return rugosity to the biofilm.

### Biophysical properties of recombinant orthologous TasA

**Figure 4:**
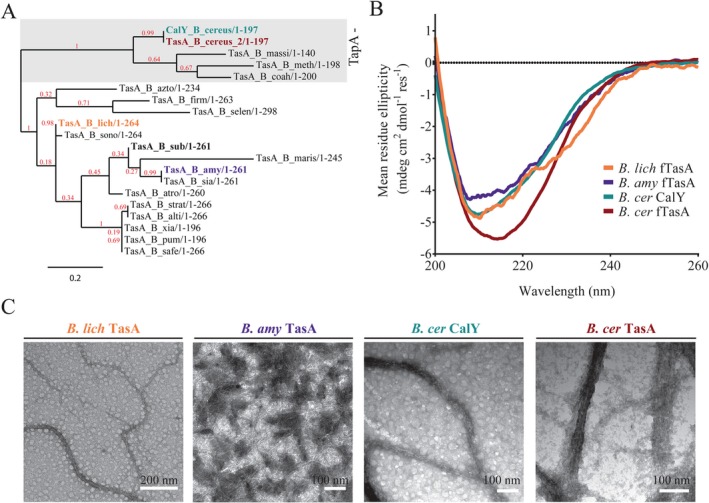
Characterisation of recombinant orthologous TasA proteins. A. The phylogenetic tree was rooted using the midpoint method with the bootstrap value (red) given as a value between 0 and 1, where 1 is a high score. Highlighted are species chosen for subsequent analysis: *B. licheniformis* (orange), *B. amyloliquefaciens* (purple), *B. cereus* TasA (red) and CalY (green). For the protein sequence alignment and abbreviations of species names alongside accession numbers see Supporting Information Fig. S5. B. Solution state circular dichroism spectra of recombinant *B. licheniformis*, *B. amyloliquefaciens*, *B. cereus* TasA and CalY. C. Transmission electron microscopy images of recombinant orthologous TasA stained with uranyl acetate show the presence of fibres that are several micron in length and vary in width from 15nm (*B. licheniformis*) to 25nm (*B. amyloliquefaciens* and *B. cereus* CalY) and 60nm (*B. cereus* TasA). A repeating unit at 4–5nm is seen for all proteins.

Using the *B. subtilis* TasA protein sequence we identified orthologous proteins from a range of *Bacillus* species. The sequences were aligned using Clustal Omega (Sievers *et al*., 2011) (Supporting Information Fig. S5) and used to generate a phylogenetic tree (Fig. 4A). Further analysis of gene synteny within the *tapA* operon revealed two distinct sub‐classes based on the presence or absence of *tapA*, as has been previously been noted for *B. cereus,* which contains two TasA paralogues but lacks *tapA* (Caro‐Astorga *et al*., 2015). Highlighted on the phylogenetic tree are *B. amyloliquefaciens* TasA, *B. licheniformis* TasA and TasA and CalY from *B. cereus* that were chosen for further analysis (Fig. 4A). Each of these proteins are predicted to encode an N‐terminal signal sequence and were used to establish: (i) whether orthologous TasA fibres assembled *in vitro* after purification of the predicted mature protein and (ii) if any fibres formed could cross‐complement the *B. subtilis* Δ*tasA sinR* deletion strain.

The quality and identity of the recombinant TasA orthologous proteins was confirmed by SDS‐PAGE (Supporting Information Fig. S6A) and mass spectrometry (Supporting Information Fig. S6B). CD spectroscopy indicated that the secondary structures of *B. licheniformis* and *B. amyloliquefaciens* TasA, and *B. cereus* CalY, were broadly similar to that of *B. subtilis* TasA, with a primary minimum at 208nm, a shoulder at ∼222nm, and a maximum below 200nm (Fig. 4B). In contrast, *B. cereus* TasA has a single broad minimum centred on ∼216nm (Fig. 4B), suggesting this protein may contain increased β‐sheet content, although both the breadth and the intensity of the minimum suggest significant remaining contribution from helical elements. TEM imaging revealed that all of the orthologous proteins spontaneously self‐assembled into fibres (Fig. 4C, Supporting Information Fig. S6C), and all showed evidence of a regular subunit repeat along the fibre axis of approximately 4–5nm. The finding that proteins corresponding to the mature region of *B. cereus* TasA and CalY form fibres *in vitro* is consistent with previous data, which revealed the presence *in vivo* of extracellular fibres dependent on *tasA* and *calY* (Caro‐Astorga *et al*., 2015) and with our findings that TapA is dispensable for TasA fibre formation *in vitro* (Fig. 3A). Through TEM imaging, we observed qualitative differences between the ability of the different proteins to form fibre bundles, with the *B. cereus* proteins TasA and CalY forming thick fibre bundles, *B. licheniformis* and *B. subtilis* TasA forming intermediate‐diameter fibre bundles and *B. amyloliquefaciens* forming a distributed mesh of thin fibres.

### *Functionality of orthologous protein fibres in* B. subtilis

**Figure 5:**
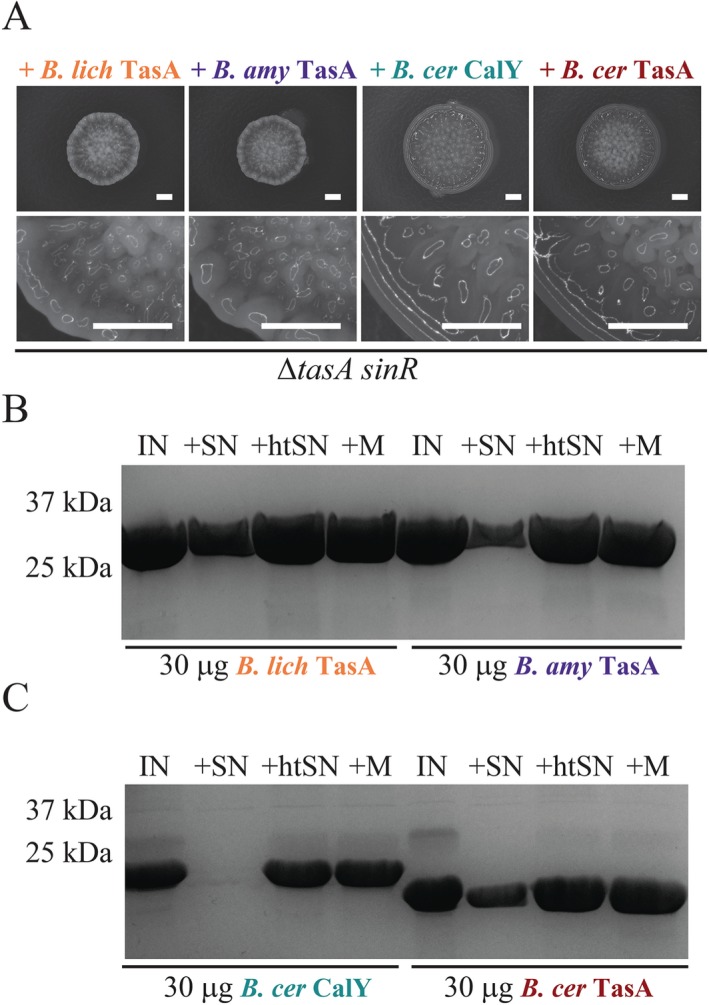
Complementation of *tasA sinR* null biofilm by recombinant orthologous TasA. A. Biofilm phenotypes shown for Δ*tasA sinR* (NRS5248) after co‐culture with 10 µg recombinant *B. licheniformis* TasA, *B. amyloliquefaciens* TasA and *B. cereus* TasA and CalY fibres. B and C. Integrity of 30 µg *B. licheniformis*, *B. amyloliquefaciens* and *B. cereus* TasA and CalY proteins incubated for 24h at 37°C analysed by SDS‐PAGE. The protein (IN) was incubated with filtered spent supernatant collected from NCIB3610 (+SN) and supernatant subjected to heat inactivation at 100°C (+htSN) alongside media only control (+M). The scale bar represents 2 mm.

To test the ability of the orthologous proteins to function in place of *B. subtilis* TasA fibres, 10 µg of each recombinant fibrous protein was exogenously added to the Δ*tasA sinR* mutant. The cells were then incubated under biofilm formation conditions. We determined that rugosity of the biofilm community could be recovered when the more closely related *B. amyloliquefaciens* and *B. licheniformis* TasA proteins were provided but not when either of the more divergent *B. cereus* proteins were supplied (Fig. 5A, Supporting Information Fig. S7A). This is in contrast to previously published data where expression of both *B. cereus calY* and *tasA*, alongside the signal peptidase *sipW*, was reported to recover biofilm formation to a *B. subtilis tasA* mutant (Caro‐Astorga *et al*., 2015). Analysis of the stability of the protein fibres after incubation with spent cell‐free culture supernatant revealed that *B. cereus* TasA fibres, like the *B. amyloliquefaciens* and *B. licheniformis* TasA fibres, were resistant to exoprotease degradation, while CalY fibres were susceptible (Supporting Information Fig. S7B and C). From our analyses of protein function we can conclude that either the interaction of TasA fibres with the *B. subtilis* matrix is dependent on the exact identity of the TasA fibres, suggesting specific molecular interactions with other matrix molecules, or that the subtle differences in the physiochemical properties of the TasA fibres may be influential in establishing rugosity in the bacterial biofilm. For example, after serially diluting the recombinant protein, and therefore shearing of the samples, recombinant *B. cereus* TasA was significantly less viscous than the equivalent samples of *B. licheniformis* and *B. subtilis* TasA (Supporting Information Fig. S7D–F) and we speculate that shearing of the samples breaks the thicker bundles observed in TEM. However, after allowing all samples to recover for 3 days, both *B. cereus* TasA and *B. licheniformis* TasA formed a gel at a lower concentration of protein than *B. subtilis* fTasA (Supporting Information Fig. S7D–F). This variability in the properties of the gels formed by the fibrous TasA orthologues may have implications for the mechanical properties of *in vivo* biofilms and the ability of one orthologue of TasA to substitute for another.

## Discussion

We have demonstrated that recombinant fibrous TasA can return rugosity to a *B. subtilis* Δ*tasA sinR* deletion strain and shares the biological functionality of native TasA purified from *B. subtilis*. Biophysical analysis indicates that these fibres are assembled as a helical arrangement of globular units that lack the characteristic ‘cross‐β’ architecture of canonical amyloid‐like fibres. The CD spectrum of the recombinant protein resembles that published previously for native TasA isolated directly from *B. subtilis* (Romero *et al*., 2010; Chai *et al*., 2013) and is suggestive of a predominantly helical secondary structure. Moreover, we have demonstrated that recombinant TasA can be rendered monomeric by the addition of a single amino acid to the N‐terminus, and that this monomeric protein shares the same secondary structure as the fibrous form. This strongly suggests that the fibres comprise a linear assembly of these monomeric units, with no large structural rearrangement, although domain‐swapping between monomers cannot be ruled out. Indeed, a repeating unit is visible along the length of the fibre axis, most clearly in the TEM images of recombinant fibres of the orthologous TasA protein from *B. cereus* where the protein subunits appear horizontally aligned across a fibre bundle, but also visible in all forms of TasA we have examined. Such a structure is not consistent with current structural models of amyloid‐like fibrils, which comprise a single continuous hydrogen‐bonded array along the long axis of the fibril. Taken together, our data indicate that TasA is unlikely to fall into the class of functional amyloid‐like fibres.

We further found that our recombinant forms of TasA did not bind either Congo Red or ThT dyes that are commonly used to assess the formation of amyloid‐like fibres. Moreover, our protein extracts from *B. subtilis* showed dye binding activity irrespective of whether TasA was present or not. Caution should be taken when inferring the formation of amyloid‐like fibres from enhanced fluorescence in the presence of ThT, which also exhibits enhanced fluorescence in the presence of globular proteins such as bovine serum albumin (Freire *et al*., 2014), human serum albumin (Sen *et al*., 2009) and acetylcholinesterase (De Ferrari *et al*., 2001); in the presence of amorphous aggregates of lysozyme and bovine serum albumin (Yang *et al*., 2015), and amorphous aggregates formed by a thrombin‐derived C‐terminal peptide (Petrlova *et al*., 2017); and in the presence of non‐amyloid wormlike aggregates of an artificial dimer of an Aβ peptide (Yamaguchi *et al*., 2010). Conversely, ThT does not exhibit enhanced fluorescence in the presence of, for example, cross‐β fibrils formed by poly‐L‐lysine (Benditt, 1986; LeVine, 1999). Congo Red is similarly promiscuous (Howie and Brewer, 2009), although the observation of green birefringence under cross‐polarisers is one of the identifying characteristics of amyloid deposits *in vivo*. Thus, Congo Red binding and enhanced ThT fluorescence should be considered only suggestive, but not indicative, of amyloid‐like fibre formation.

The widespread nature of functional amyloid fibres in bacterial biofilms has been hypothesized, and a well‐characterised example is the curli fibres of *E. coli*, *Enterobacter cloacae*, and *Salmonella* spp (Evans and Chapman, 2014). These show a CD spectrum, dye‐binding behaviour, enhanced stability and proteolytic insensitivity that are consistent with an amyloid‐like β‐sheet structure, but solid‐state NMR data suggests an architecture comprising stacked β‐helical subunits (Shewmaker *et al*., 2009), a structural motif commonly employed by bacteria (Kajava and Steven, 2006). Many amyloid‐like fibres formed *in vitro* from proteins associated with disease show an in‐register parallel cross‐β arrangement (Margittai and Langen, 2008); recently however native Tau filaments extracted from the brain of an Alzheimer's Disease patient have been demonstrated to form an elaborate mixed β‐helix/cross‐β structure formed of in‐register, parallel β‐strands (Fitzpatrick *et al*., 2017). Thus, both cross‐β and β‐helix architectures may be characteristic of amyloid fibres, and curli fibres may still be considered as ‘amyloid‐like’.

Making the correct distinction between amyloid‐like and non‐amyloid fibrous proteins is more than a semantic argument: a number of papers have drawn a link between functional amyloid‐like fibres formed by bacteria and their relevance to human disease (Epstein and Chapman, 2008; Chai *et al*., 2013; Evans and Chapman, 2014), for example, in the determination of the mechanistic details of self‐assembly, or in the possible discovery of new therapeutics. As the amyloid‐like fibre macrostructure is thought to be a ‘generic’ property deriving from the chemical structure of the polypeptide backbone that is common to all proteins and peptides – and thus to a large extent independent of primary sequence, although this will influence overall fibre morphology – small drug molecules that target the generic amyloid fold may have widespread applicability in a number of devastating human diseases. Thus it is important to make the distinction between non‐amyloid fibrous assemblies and amyloid‐like fibres appropriately.

The fibrous nature of TasA likely imparts mechanical rigidity to the biofilm, thereby restoring the highly wrinkled architecture characteristic of the *ΔtasA sinR* deletion strain. As indicated above it is unclear why neither fTasA nor nTasA(+) can recover biofilm architecture to the single *tasA* deletion and furthermore, why expression of a *sipW‐tasA* construct is required for genetic complementation. Since SinR has pleiotropic roles in biofilm formation (Vlamakis *et al*., 2013; Cairns *et al*., 2014) it may be that overproduction of the biofilm polysaccharide compensates for the loss of native regulation that intricately controls native TasA production in space and time (Vlamakis *et al*., 2008). Our results also indicate that when in a fibrous form, TasA does not require the TapA protein to fulfil its function, which contradicts previous reports suggesting that TapA is an accessory protein required for correct TasA assembly and localisation (Romero *et al*., 2011). Therefore the role played by TapA in biofilm formation, while evidently essential (Chu *et al*., 2006), is unclear. It may be that while TapA is not essential for TasA fibre formation *in vitro*, it functions as a chaperone *in vivo* to aid the transition of monomeric TasA into a fibrous state. This hypothesis is consistent with the overall reduction in the level of TasA and the corresponding reduction in the number of TasA fibres observed in the *tapA* mutant (Romero *et al*., 2011). Moreover, it is consistent with the demonstration that monomeric TasA, but not fibrous TasA, is susceptible to degradation by the extracellular proteases.

A non‐amyloid‐like structure for TasA is possibly beneficial in the context of the *B. subtilis* biofilm; amyloid‐like self‐assembled fibres are very stable, with curli fibres, for example, requiring treatment with concentrated acid solutions to drive disassembly (Chapman *et al*., 2002). Curli fibres also appear to form a brittle matrix which, once fractured, does not recover (Serra *et al*., 2013). In contrast, we have shown that the gelation properties of fibrous TasA solutions recover after shear (Supporting Information Fig. S5D–F), suggesting that *in vivo* the biofilm matrix could be remodelled in response to mechanical environmental perturbations. The TasA fibres may also be in equilibrium with the monomeric form of the protein, which would allow dynamic restructuring of the biofilm in response to environmental changes. As the fibrous form of the protein confers protection against degradation by extracellular proteases whereas the monomeric protein is degraded, an appropriate secretion of monomeric protein and/or proteases could provide dynamic control of biofilm elasticity and structure.

## Experimental procedures

### Growth conditions

*E. coli* and *B. subtilis* were routinely grown in Lysogeny Broth (LB) media (10g NaCl, 5g yeast extract and 10g tryptone per litre) or plates supplemented with the addition of 1.5% (w/v) select agar (Invitrogen). Samples were grown at 37°C unless stated otherwise. When required, antibiotics were used at the following concentrations: ampicillin (100 μg ml^−1^), spectinomycin (100 μg ml^−1^) and chloramphenicol (5 µg ml^−1^). For biofilm assays MSgg minimal media was used (5mM KH_2_PO_4_ and 100mM MOPS at pH 7 supplemented with 2mM MgCl_2_, 700 µM CaCl_2_, 50 µM MnCl_2_, 50 µM FeCl_3_, 1 µM ZnCl_2_, 2 µM thiamine, 0.5% glycerol, 0.5% glutamate). When appropriate isopropyl β‐D‐1‐thiogalactopyranoside (IPTG) was added at the indicated concentration. For protein production auto‐induction media (6g Na_2_HPO_4_, 3g KH_2_PO_4_, 20g Tryptone, 5g yeast extract, 5g NaCl, 10ml 60% v/v glycerol, 5ml 10% w/v glucose and 25ml 8% w/v lactose per litre at a 1:1000 volume ratio (supplemented with 100 µg/ml ampicillin)) was used (Studier, 2005).

### Strain construction

A complete list of *E. coli* and *B. subtilis* strains used in this study can be found in Supporting Information Table S1. Plasmids and primers are detailed in Supporting Information Tables S2 and S3 respectively. All *B. subtilis* strains used for physiological assays were derived from the wild‐type laboratory isolate NCIB3610 and constructed using standard protocols. SSP1 phage transductions for DNA transfer into *B. subtilis* NCIB3610 were carried out as previously described (Verhamme *et al*., 2007).

### Plasmid construction and mutagenesis

Construction of an in‐frame *tasA* deletion in NCIB3610 was achieved using the pMiniMAD (Patrick and Kearns, 2008) temperature sensitive allelic replacement vector pNW1448 (pMiniMAD‐Δ*tasA*). The plasmid was constructed by PCR amplification of two fragments: the 511 bp upstream of *tasA* including the first 6 bp of *tasA* coding sequence and a second fragment covering the last 3bp of the *tasA* coding sequence, the stop codon and the 512 bp downstream using primer pairs NSW2005/NSW2006 and NSW2007/NSW2008 respectively. The PCR fragments were each digested with SalI/EcoRI and simultaneously ligated into the pMiniMAD plasmid that was digested with the same restriction sites to yield plasmid pNW1448. Plasmid pNW1448 was introduced into 168 and then transferred to NCIB3610 using phage transduction. The *tasA* deletion was introduced into the *B. subtilis* chromosome using the method described previously (Arnaud *et al*., 2004). After homologous recombination and selection for loss of the pMiniMad plasmid, two morphologically distinct isolates carrying the desired deletion in *tasA* were identified. Whole genome sequencing (see below) was used to genotype the isolates in an unbiased manner. Analysis of single nucleotide polymorphisms (Supporting Information Table S4) revealed one strain carried a short duplication of the *sinR* coding region effectively yielding a *ΔtasA sinR* double mutant (NRS5248) while the other was a single Δ*tasA* strain (NRS5267).

The *tapA* in frame deletion was generated via the pMAD protocol as above, with amplification of the 395 bp upstream fragment using primers NSW1308 and NSW1332 and 641 bp downstream fragment using primers NSW1333 and NSW1334. The two PCR fragments were digested BamHI/SalI and EcoRI/SalI respectively and ligated into the intermediate plasmid pUC19 yielding pNW686, and was subsequently moved into pMAD to generate pNW685. Plasmid pNW685 was introduced into *B. subtilis* 168, generating strain NRS3789, and transferred to NCIB3610 using phage transduction. The same 168 strain was used to generate the Δ*tapA* Δ*tasA sinR* strain by transferring via phage to Δ*tasA sinR* (NRS5248).

Genetic complementation of Δ*tasA* and Δ*tasA sinR* was achieved by PCR amplification of the *tasA* (using primers NSW1857 and NSW1858) and *sipW‐tasA* (using primers NSW2218 and 2219) regions from NCIB3610. Both were cut using SalI/SphI restriction enzymes and ligated into the pDR183 (pNW1434) and pDR110 (pNW1432 and pNW1619) vector. Plasmid pNW1434 pDR183 was introduced to 168 and transferred to Δ*tasA sinR* (NRS5255). Plasmids pNW1432 and pNW1619 were introduced into 168 and transferred to Δ*tasA* (NRS5276) and Δ*tasA sinR* (NRS5248) using phage transduction.

Genetic complementation of the triple Δ*tapA* Δ*tasA sinR* (NRS5749) mutant was performed using the whole *tapA‐sipW‐tasA* operon which was amplified from NCIB3610 using primers NSW1896 and NSW2219, cut SalI/SphI and ligated into pDR110 to generate pNW1804 which was introduced by phage transduction via 168 at the ectopic *amyE* location on the chromosome.

Protein purification was achieved using GST fusion constructs. The *tasA* overexpression plasmid pNW1437 (pGex‐6‐P‐1‐TEV‐*tasA*_(28–261)_) was generated by amplifying the *tasA*_(28–261)_ coding region from *B. subtilis* NCIB3610 genomic DNA using primers NSW660 and NSW661 and insertion into the vector pGEX‐6P‐1 cleaved BamHI/XhoI yielding the vector pNW543. The TEV protease cleavage site was next introduced by site‐directed mutagenesis using primers NSW1892 and NSW1893 to give pNW1437. Amino acids were introduced at the N‐terminal end of *tasA* also by site‐directed mutagenesis; primer pairs are indicated in Supporting Information Table S2. The constructs used to purify the TasA orthologue proteins were generated in a similar manner from genomic DNA isolated from *B. cereus* ATCC14579, *B. licheniformis* ATCC14580 and *B. amyloliquefaciens* FZB42 and likewise primers used for amplification are detailed. The plasmids were used to transform BL21 (DE3) *E. coli* strain for protein production.

### Genome sequencing

Whole genome sequencing and bioinformatics analysis of strains NCIB3610, NRS5248 and NRS5267 was conducted by MicrobesNG (http://microbesng.uk) which is supported by the BBSRC (grant number BB/L024209/1). Three beads were washed with extraction buffer containing lysozyme and RNase A, incubated for 25 min at 37°C. Proteinase K and RNaseA were added and incubated for 5 min at 65°C. Genomic DNA was purified using an equal volume of SPRI beads and resuspended in EB buffer. DNA was quantified in triplicates with the Quantit dsDNA HS assay in an Eppendorf AF2200 plate reader. Genomic DNA libraries were prepared using Nextera XT Library Prep Kit (Illumina™, San Diego, USA) following the manufacturer's protocol with the following modifications: 2 ng of DNA instead of one were used as input, and PCR elongation time was increased to 1 min from 30s. DNA quantification and library preparation were carried out on a Hamilton Microlab STAR automated liquid handling system. Pooled libraries were quantified using the Kapa Biosystems Library Quantification Kit for Illumina on a Roche light cycler 96 qPCR machine. Libraries were sequenced on the Illumina HiSeq using a 250 bp paired end protocol. Reads were adapter trimmed using Trimmomatic 0.30 with a sliding window quality cut‐off of Q15 (Bolger *et al*., 2014). De novo assembly was performed on samples using SPAdes version 3.7 (Bankevich *et al*., 2012) and contigs were ordered using Abacas (Assefa *et al*., 2009) and annotated using Prokka 1.11 (Seemann, 2014). Reads were aligned to the reference 168 genome (accession number: NZ_CM000487.1) using BWA‐Mem 0.7.5 and processed using SAMtools 1.2 (Li *et al*., 2009). Variants were called using VarScan 2.3.9 with two thresholds, sensitive and specific, where the variant allele frequency is greater than 90% and 10% respectively. The effects of the variants were predicted and annotated using SnpEff 4.2 (Koboldt *et al*., 2009) (Supporting Information Table S4).

### Protein production and purification

The pGEX‐6‐P‐1 GST‐gene fusion system was used in the *E. coli* BL21 (DE3) strain for protein production (GE Healthcare™) (Studier and Moffatt 1986). After the required plasmid was introduced into BL21(DE3), a 5ml LB culture (supplemented with 100 µg/ml ampicillin) was grown overnight at 37°C and used to inoculate 1l of auto‐induction media at 1/1000 dilution. The cultures were incubated at 37°C with 130rpm shaking until optical density at 600nm was approximately 0.9 at which point the temperature was lowered to 18°C and cultures were grown overnight. Cells were harvested by centrifugation at 4000g for 45 min and the cell pellet was suspended in 25ml of purification buffer (25mM Tris‐HCl, 250mM NaCl, pH 7.5) supplemented with Complete EDTA‐free Protease inhibitor (Roche) then lysed using an Emulsiflex cell disrupter (Avestin™) with 3 passes made at ∼15,000 psi or sonication at 25% for 6 min. Cell debris was removed by centrifugation at 27,000g for 35 min. The supernatant was removed and added to 450 µl of Glutathione Sepharose 4B beads (GE Healthcare™) and incubated on a roller for 3h at 4°C. The protein‐bead mix was loaded onto disposable gravity flow columns (Bio‐Rad™) and washed three times with 25ml of purification buffer. Beads were collected from the column and suspended in 25ml of purification buffer supplemented with 1mM DTT and 0.5mg TEV protease and incubated on roller at 4°C overnight. The flow‐through was then added to 300 µl GST beads and 250 µl Ni‐NTA beads (Qiagen™) and incubated for 2h at 4°C. Final pass through column removes beads and flow‐through is concentrated using 10kDa Vivaspin™. For biophysical experiments performed at Edinburgh University, buffer exchange into 25mM phosphate buffer (pH 7) was performed using same concentrators. Purity was determined by SDS‐PAGE and molecular mass determined by loading 80 µg onto a qTOF liquid chromatography mass spectrometry performed by the FingerPrints Proteomics Facility at the University of Dundee.

### *Native extraction from* B. subtilis

Method adapted from (Romero *et al*., 2010). Briefly, cells from the *eps sinR* double mutant and *eps sinR tasA* triple mutant were grown in 1 L Msgg at 37° at 130rpm for 20h from an OD_600_ of 0.02. Cells were pelleted at 5000g for 30 min and the media discarded. Cells were centrifuged twice with 25ml extraction buffer [5mM KH_2_PO_4_, 2mM MgCl_2_, 100mM MOPS (pH 7), 1M NaCl with Roche Protease Inhibitor cocktail] and the supernatant filtered through a 0.4 µm filter. Ammonium sulphate was added to make 30% in final volume and incubated with stirring at 4°C for 1h. The supernatant was then centrifuged at 20,000g for 10 min to remove precipitated proteins and dialysed twice in 5L 25 mM phosphate buffer (pH 7) at room temperature for 1h each and then 4°C overnight.

### Insulin fibre preparation

Insulin fibres were prepared as previously described (Shammas *et al*., 2011). A 0.05mg ml^−1^ solution of insulin fibres were analysed by CD spectroscopy.

### Transthyretin fibre preparation

TTR fibres were prepared as previously described (Schor *et al*., 2015). Briefly, 0.8mg of the peptide was dissolved in 200 µL 25mM phosphate buffer (pH 7) for *ex vivo* complementation and 20% (w/v) acetonitrile (pH 5) for X‐ray diffraction pattern collection. Sample was sonicated for 10 min and combined with 10 µl of TTR seeds and incubated at 60°C for 5h.

### Biofilm phenotypes, ex vivo complementation and protein collection

To characterise biofilm phenotype samples were set up as detailed previously (Branda *et al*., 2001). Briefly, 10 µl of LB culture grown to mid‐exponential phase was spotted onto solidified MSgg media and incubated for 2 days at 30°C. The resultant colonies were imaged using a Leica MZ16 stereoscope. For *ex vivo* complementation, 10 µg of recombinant protein, 30 µg native extract or 10 µl TTR where indicated was pipetted with cells immediately prior to spotting. To release all biofilm proteins for subsequent analysis, the biofilm was resuspended in 500 µl BugBuster Master Mix (Novagen) followed by sonication and agitation for 20 min at room temperature. Insoluble debris was removed by centrifugation at 17,000g for 10 min at 4°C.

### SDS‐PAGE

SDS‐polyacrylamide gel electrophoresis (PAGE) was performed using 10 µg of purified TasA protein and 4X loading buffer (6.2g SDS, 40ml 0.5 M Tris pH 6.8, 6.4ml 0.1 M EDTA, 32ml 100% glycerol, 1mg Bromophenol blue). Samples were heated at 99°C for 5 min prior to loading on the gel and were run on a standard 14% polyacrylamide SDS‐PAGE at 200 V for 60 min, before staining with InstantBlue (Expedeon™).

### Immunoblot analysis

Samples were separated by SDS‐PAGE and transferred onto a PVDF membrane (Millipore™) by electroblotting at 100 mA for 75 min. The membranes were blocked with 3% (w/v) milk in 1xTBS overnight at 4°C with shaking followed by 1h incubation with primary antibody (TasA (1:25,000 v/v) as indicated) diluted in 3% (w/v) milk in 1x TBS. Production of the TasA antibody has been previously described (Ostrowski *et al*., 2011). This was followed by 3 washes of 10 min each with 1x TBS and 2% (v/v) Tween20 and subsequent 45 min incubation with goat anti‐rabbit conjugated secondary antibody (1:5000 v/v) (Pierce™). Membrane was washed 3 times for 10 mins with TBST then developed by ECL incubation and exposing to X‐ray film (Konica™) using the Medical Film Processor SRX‐101A (Konica™). This is with the exception of the data shown in Fig. 4C which was developed as detailed above and visualised using GeneGnomeXRQ (Synegene™).

### Size‐exclusion chromatography

Monomeric TasA was examined by size‐exclusion chromatography using either Superdex 5/150 or 10/300 GL increase column as indicated (GE Healthcare) on an ÄKTA FPLC system using 25mM Tris‐HCl, 250mM NaCl, pH 7 buffer. The column was calibrated using conalbumin (75000 Da), ovalbumin (44,000 Da), carbonic anhydrase (29,000 Da), ribonuclease A (13,700 Da) and aprotinin (6500 Da) and void volume was calculated using blue dextran 2000 (GE Healthcare).

### Exoprotease stability

PY79, PY79 Δ6 and PY79 Δ7 and/or NCIB3610 were grown to an OD_600_ of ∼2.5in 25 ml MSgg growth media at 37°C with 130RPM shaking overnight. The cultures were normalised to same OD_600_ and 5ml was centrifugation at 3750 *g* for 15 min at 4°C to pellet cells. The culture supernatant was collected and filtered through a 0.22 µM filter (Milipore) to remove residual cells. Aliquots of the culture supernatant generated by NCIB3610 and PY79 were heated inactivated at 100°C for 10 min as required. A 15 µl of each cell‐free culture supernatant was incubated with 30 µg recombinant protein alongside a media only‐control at 37°C for 24h. The integrity of the protein was analysed by SDS‐PAGE alongside a non‐incubated sample of recombinant protein as a loading control.

### Protein sequence alignment

TasA orthologues were identified by BLASTP (Altschul *et al*., 1990, 1997) using the protein sequence of TasA from *B. subtilis* as the query. TasA protein sequences were aligned using Clustal Omega with the default settings (Sievers *et al*., 2011). The aligned sequences were imported and manually coloured for homology as indicated in the legend in Microsoft Word. The signal sequences were predicted using the SignalP v4.1 server and are indicated by underline (Petersen *et al*., 2011). A maximum likelihood tree was calculated from the Clustal Omega alignment using the phylogeny.fr platform (Dereeper *et al*., 2008), Gblocks was used to eliminate divergent and poorly aligned segments for tree construction (Castresana, 2000). The tree was estimated using the PhyML algorithm (Guindon and Gascuel, 2003) with mid‐point rooting, using a WAG substitution model (Whelan and Goldman, 2001) and bootstrapping procedure set to 100 replicates. The outputted tree was visualised using TreeDyn (Chevenet *et al*., 2006).

### Protein precipitation of TasA for mass spectrometry

A strain carrying an IPTG inducible copy of the *tasA* gene (NRS5313) in a Δ*tasA* background was grown in 200ml Msgg at 37°C until OD_600_ of 1 in the presence of 1mM IPTG. The culture supernatant was collected and separated from cell fraction by centrifugation at 5000 *g* for 30 min at 4°C with iterative removal of the supernatant into a fresh tube for 4 rounds. 40ml of the clarified supernatant was precipitated overnight with 6.25% (w/v) trichloroacetic acid (Sigma^TM^) at 4°C and the precipitated proteins were recovered by centrifugation as before. The protein pellet was washed 5 times with 1ml ice‐cold dH_2_O and air dried for 1h (protocol modified from Cianfanelli *et al*. 2016). The protein pellet was suspended in 50 µl 2x Laemmli buffer and separated by SDS‐PAGE on a 14% gel alongside *in vitro* purified fTasA protein as a size marker. The section of the lane at the position expected to contain mature TasA was excised and analysed by mass spectrometry.

### Mass spectrometry

Samples were processed prior to overnight (16h) trypsin digestion (Modified Sequencing Grade, Pierce). Peptides extracted from gel and dried in SpeedVac (Thermo Scientific^TM^). Peptides were re‐suspended 50 µl 1% formic acid, centrifuged and transferred to HPLC vial. A 15 µl of each sample was typically analysed on the system. The peptides from each fraction were separated using a mix of buffer A (0.1% formic acid in MS grade water) and B (0.08% formic acid in 80% MS grade CH_3_CN). The peptides from each fraction were eluted from the column using a flow rate of 300 nl/min and a linear gradient from 5% to 40% buffer B over 68 min. The column temperature was set at 50°C. The Q Exactive HF Hybrid Quadrupole‐Orbitrap Mass Spectrometer was operated in data dependent mode with a single MS survey scan from 335 to 1800m/z followed by 20 sequential *m/z* dependent MS2 scans. The 20 most intense precursor ions were sequentially fragmented by higher energy collision dissociation (HCD). The MS1 isolation window was set to 2.0 Da and the resolution set at 60,000. MS2 resolution was set at 15,000. The AGC targets for MS1 and MS2 were set at 3e^6^ ions and 5e^5^ ions respectively. The normalized collision energy was set at 27%. The maximum ion injection times for MS1 and MS2 were set at 50 ms and 100 ms respectively. Exactive HF Hybrid Quadropole .RAW data files were extracted and converted to mascot generic files (.mgf) using MSC Convert. Extracted data then searched against the Local peptide database containing the relevant TasA sequence using the Mascot Search Engine (Mascot Daemon Version 2.3.2).

### Fibre formation and X‐ray diffraction

To prepare samples for X‐ray diffraction, 5 µl of recombinant at ∼ 5mg ml^−1^ fTasA was suspended between two borosilicate glass capillaries (Harvard Apparatus) and allowed to dry (Makin & Serpell, 2005). The dried fibres were mounted onto a Rigaku M007HF X‐ray generator equipped with a Saturn 944HG+ CCD detector, and images collected with 60s exposures at room temperature. Diffraction patterns were inspected using Ipmosflm CCP4} and then converted to TIFF format. CLEARER (Sumner Makin *et al*., 2007) was used to measure the diffraction signal positions.

### Circular dichroism spectroscopy

All Circular dichroism (CD) measurements were performed using a Jasco J‐810 spectropolarimeter. Solution‐state samples were measured at a protein concentration of 0.2mg ml^−1^ (in 25mM phosphate buffer) in a 0.1cm quartz cuvette. A scan rate of 50nm s^−1^ was used, with a data pitch of 0.1nm and a digital integration time of 1s. Twenty scans were accumulated and averaged to produce the final curve.

### Transmission electron microscopy (TEM) imaging

A 5 μl droplet of 0.02mg ml^−1^ protein solution was pipetted onto a carbon‐coated copper grid (TAAB Laboratories Equipment Ltd) and left for 4 min before being wicked away from the side with filter paper. Subsequently, a 5 μl droplet of 2% (w/v) uranyl acetate was pipetted onto the grid and left for 3 min before being wicked away from the side with filter paper. The stained grids were imaged using a Philips/FEI CM120 BioTwin transmission electron microscope and ImageJ software was used for image analysis.

### Thioflavin T binding kinetics

Protein samples were diluted to 3mg ml^−1^ in 25mM phosphate buffer. 200 μl of protein was added into the wells of a Corning NBS 96‐well plate (Corning 3641). ThT was added to a final concentration of 20 μM. The plates were sealed with a transparent film and put into a BMG Fluostar plate reader at 37°C as indicated. Measurements of ThT fluorescence were taken every 5 min for a period of 8h for mTasA and fTasA, the median of these values in represented in Supporting Information Fig. S1G. For nTasA(+), nTasA(–) and controls, only a single read was taken.

### Congo red binding assay

A stock solution of 2mg ml^−1^ Congo Red (Sigma‐Aldrich 75768) was prepared in phosphate buffer and filtered three times using a 0.22 μm syringe filter (Millipore). A 2mg ml^−1^ bovine insulin (Sigma‐Aldrich I5500) was prepared in MilliQ water adjusted to pH 1.6 using concentrated HCl. The insulin sample was incubated overnight at 60°C. A 60 µl of each protein sample was added to a cuvette containing 1ml of buffer and 10 µl of the Congo Red stock solution. The samples were then allowed to incubate at room temperature for 30 min. A control spectrum containing only Congo Red was measured where 10 µl of the Congo Red stock solution was added to 1ml of buffer plus an additional 60 μl of buffer (to match the amount of protein added to each cuvette). Since the nTasA(±) samples contained multiple components, a UV‐vis spectrum (Cary 1E spectrophotometer) from 800 to 200nm was measured and the relative absorbance peaks at 280nm was used to ensure equal amounts of protein were measured between the two samples. The Congo Red spectra were acquired over a wavelength range of 400–700nm.

### Mean square displacement via bead tracking

A 1 μl aliquot of carboxylate‐modified polystyrene, fluorescent yellow‐green latex beads with a mean particle size of 1 μm (Sigma‐Aldrich, L4655) was diluted into 1ml of phosphate buffer. A 5 μl of this stock solution was added to the protein solution and gently mixed to disperse the particles. A 80 μl of the bead and protein solution was placed on a cavity slide (Brand GmBH, 0.6–0.8mm depth) and sealed with a coverslip using nail varnish. Movies of the motion of the particles were taken using a Nikon Eclipe Ti microscope equipped with a Hamamatsu Orca‐Flash 4.0 CCD camera. Images were acquired using μ‐manager software at a framerate of 10 fps (Edelstein *et al*., 2010). Movies were then analysed using TrackPy (available from github.com/soft‐matter/trackpy).

## Contributions

Conceived and designed the experiments: CE, EE, RG, CEM, RJM, MS, NSW; Performed the experiments: KMB, LC, CE, EE, PKF, RG, CEM, RJM, MS, TS; Contributed new analytical tools: CE, EE, RG, TS; Analysed the data: CE, EE, CEM, RJM, MS, LCS, NSW; Wrote the paper: EE, RJM, CEM, MS, NSW.
