# Supplementary material for: Formation of functional, non‐amyloidogenic fibres by recombinant Bacillus subtilis TasA
Source: Mol Microbiol. 2018 Nov 16;110(6):897–913. doi: 10.1111/mmi.13985 (PMC6334530; doi:10.1111/mmi.13985)
Supplement: Supplementary file 1 [file MMI-110-897-s001.pdf]

Supplemental Data

Formation of functional, non-amyloidogenic fibres by recombinant *Bacillus subtilis* TasA

Elliot Erskine[1*], Ryan J Morris [2*], Marieke Schor [2*], Chris Earl [1], Rachel M. C. Gillespie [1], Keith Bromley [2] Tetyana Sukhodub[1], Lauren Clark[2], Paul K. Fyfe[1], Louise C. Serpell [3], Nicola R. Stanley-Wall [1#], Cait E. MacPhee[2#]

[1] Division of Molecular Microbiology, School of Life Sciences, University of Dundee, Dundee, UK DD1 5EH

[2] James Clerk Maxwell Building, School of Physics, University of Edinburgh, The Kings Buildings, Mayfield Road, Edinburgh EH9 3JZ

[3] School of Life Sciences, University of Sussex, Falmer, BN1 9QG

*These authors contributed equally and are listed alphabetically

# These authors contributed equally

Running Title: Functional recombinant non-amyloidogenic TasA fibres

To whom correspondence should be addressed:

Prof. Cait MacPhee

James Clerk Maxwell Building, School of Physics and Astronomy, University of Edinburgh, Edinburgh EH9 3JZ, United Kingdom

Email: cait.macphee@ed.ac.uk

OR

Prof. Nicola R. Stanley-Wall

Division of Molecular Microbiology, School of Life Sciences, University of Dundee, Dundee DD1 5EH.

Email: n.r.stanleywall@dundee.ac.uk

Keywords: **TasA fibres, *Bacillus subtilis*, biofilm matrix, functional amyloid, amyloid-like fibres**

Contributions. Conceived and designed the experiments: CE, EE, RG, CEM, RJM, MS, NSW; Performed the experiments: KB, LC, CE, EE, PKF, RG, CEM, RJM, MS, TS; Contributed new analytical tools: CE, EE, RG, TS; Analysed the data: CE, EE, CEM, RJM, MS, LCS, NSW; Wrote the paper: EE, RJM,CEM, MS, NSW.

Table S1. *B. subtilis* and *E. coli* strains used in this study

Strain	Relevant genotype /Description	Source / Construction [b,c]
MC1061	*E. coli F'lacIQ lacZM15 Tn10* (*tet*)	*E. coli* Genetic Stock Centre
BL21	F– *ompT hsdSB*(rB–, mB–) *gal dcm* (DE3)	(Studier and Moffatt, 1986)
NCIB3610	Prototrophic wild-type strain	BGSC
168	*trpC2*	BGSC
NRS5048	168 pMiniMAD-Δ*tasA*	pNW1448 → 168
NRS5267	NCIB3610 Δ*tasA*	SPP1 NRS5048 → 3610
NRS5276	NCIB3610 Δ*tasA lacA*::P_{IPTG}-*tasA*	pNW1434 → NRS5267
NRS5313	NCIB3610 Δ*tasA amyE*::P_{IPTG}-sipW-*tasA*	pNW1619 → NRS5267
NRS5316	NCIB3610 Δ*tasA amyE*::P_{IPTG}-$sipW_{TAA}$-*tasA*	pNW1631 → NRS5267
NRS5248	NCIB3610 Δ*tasA* $sinR_{(Phe65\_Iso71dup)}$	SPP1 NRS5048 → 3610
NRS5255	NCIB3610 Δ*tasA sinR amyE::*P_{IPTG}-*tasA* (spc)	pNW1432 → NRS5248
NRS1661	168 *epsA*::pBL584Φ(P_{IPTG}-*epsA*) (cml)	pBL584 → 168
NRS5421	NCIB3610 Δ*tasA sinR epsA*::pBL584Φ(P_{IPTG}-*epsA*) (cml)	SPP1 NRS1661 → NRS5248
NRS3789	168 containing pNW685	pNW685 → 168
NRS5749	NCIB3610 Δ*tapA* Δ*tasA sinR*	SPP1 NRS3789 → NRS5248
NRS5760	168 *amyE::*P_{IPTG}*-tapA-sipW-tasA-lacI (spc)*	pNW1804 → 168
NRS5763	NCIB3610 Δ*tapA* Δ*tasA sinR amyE::*P_{IPTG}*-tapA-sipW-tasA-lacI (spc)*	SPP1 NRS5760 → NRS 5749
NRS2450	NCIB3610 *eps(A-O)::tet*	(Ostrowski *et al.*, 2011)
NRS1235	168 *sinR::cat*	NSW laboratory stocks
NRS5422	*eps(A-O)::tet sinR::cat*	SPP1 NRS1235 → NRS2449
NRS1858	168 *sinR::kan*	(Kiley and Stanley-Wall,
NRS5931	*eps(A-O)::tet sinR::kan tasA::spc*	SPP1 1858 → NRS2450
PY79 Δ7	PY79 *nprE aprE epr mpr nprB vpr bpr*	BGSC KO7 (1A1133)
PY79 Δ6	PY79 *nprE aprE epr mpr nprB vpr*	BGSC KO6

1. Drug resistance cassettes are indicated as follows: *spc*, spectinomycin, *amp*: ampicillin, cml: chloramphenicol, kan:: kanamycin
2. BSGC represents the *Bacillus* genetic stock centre.
3. The direction of strain construction is indicated with plasmid DNA or phage (SPP1) (→) recipient strain.

Table S2 Plasmids used in this study

Plasmid	Description	Source
**pDR111**	*B. subtilis* integration vector for IPTG-induced expression	Britton *et al*., 2002
**pGEX-6P-1**	Vector for overexpression of GST-fused proteins	GE Healthcare
**pMiniMAD**	Temp sensitive allelic replacement vector	Patrick & Kearns., 2008
**pNW1448**	pMinimad Δ*tasA*	This work
**pNW1619**	pDR110 P_{IPTG}-*sipW-tasA* (spc)[1]	This work
**pNW1437**	pDR110 P_{IPTG}-*tasA* (spc)[1]	This work
**pNW1631**	pDR110 P_{IPTG}-$sipW_{TAA}$-*tasA*	This work
**pNW685**	pMAD Δ*tapA*	This work
**pNW1432**	pDR110 P_{IPTG}-*tasA* (spc)[1]	This work
**pNW1434**	pDR183 P_{IPTG}-*tasA* (erm)[1]	This work
**pNW543**	pGEX-6P-1- $tasA_{(28\text{-}261)BS}$ (amp)[1,2]	This work
**pNW1437**	pGEX-6P-1-TEV-$tasA_{(28\text{-}261)BS}$ (amp)[1,2]	This work
**pNW1080**	pGEX-6P-1-TEV-ser-$tasA_{(28\text{-}261)BS}$ (amp)[1,2]	This work
**pNW1082**	pGEX-6P-1-TEV-ala-$tasA_{(28\text{-}261)BS}$ (amp)[1,2]	This work
**pNW1083**	pGEX-6P-1-TEV-glu-$tasA_{(28\text{-}261)BS}$ (amp)[1,2]	This work
**pNW1084**	pGEX-6P-1-TEV-lys-$tasA_{(28\text{-}261)BS}$ (amp)[1,2]	This work
**pNW1085**	pGEX-6P-1-TEV-phe-$tasA_{(28\text{-}261)BS}$ (amp)[1,2]	This work
**pNW1606**	pGEX-6P-1-TEV-$tasA_{(28\text{-}264)BL}$ (amp)[1,2]	This work
**pNW1608**	pGEX-6P-1-TEV-$tasA_{(28\text{-}261)BA}$ (amp)[1,2]	This work
**pNW1096**	pGEX-6P-1-TEV-$tasA_{(28\text{-}197)BC1}$ (amp)[1,2]	This work
**pNW1616**	pGEX-6P-1-TEV-$tasA_{(28\text{-}197)BC2}$ (amp)[1,2]	This work
**pBL584**	*epsA*::pBL584Φ(P*spachy*-*epsA*)-*cat* (cml)[1]	(Terra *et al.*, 2012)
**pNW1804**	pDR110- *tapA-sipW-tasA*	This work

1. Drug resistance cassettes are indicated as follows: *spc*, spectinomycin, *amp*: ampicillin, cml: chloramphenicol, erm: erythromycin

2. Species abbreviations: BS (*B. subtilis*), BL (*B. licheniformis*), BA (*B. amyloliquefaciens*), BC1 (*B. cereus* TasA), BC2 (*B. cereus* CalY). The amino acids of the protein sequence encoded by the construct are detailed in brackets.

Table S3. Primers used in this study.

Primer	Sequence (5'-3')	Purpose	Plasmid
NSW2005	GCATGTCGACAGTTCTGTCAGGTTCGATGG	Upstream *tasA* region for deletion	pNW1448
NSW2006	GCATGGATCCACCCATGGTAAGCTCCCCTTTTA		
NSW2007	GCATGGATCCAATTAATAACAGCAAAAAAAAGAGACGGCCC	Downstream *tasA* region for deletion	
NSW2008	GCATGAATTCAAGTCTGCTCATCCTGGTCC		
NSW2100	*GAAAATTTATATTTTCAA***TCC**GCATTTAACGACATTAAA	Addition of serine to pNW1437	pNW1080
NSW2101	TTTAATGTCGTTAAATGC**GGA***TTGAAAATATAAATTTTC*		
NSW2110	*GAAAATTTATATTTTCAA***GCT**GCATTTAACGACATTAAA	Addition of Alanine to pNW1437	pNW1082
NSW2111	TTTAATGTCGTTAAATGC**AGC***TTGAAAATATAAATTTTC*		
NSW2112	*GAAAATTTATATTTTCAA***TTT**GCATTTAACGACATTAAA	Addition of Phenylalanine to pNW1437	pNW1085
NSW2113	TTTAATGTCGTTAAATGC**AAA***TTGAAAATATAAATTTTC*		
NSW2114	*GAAAATTTATATTTTCAA***AAA**GCATTTAACGACATTAAA	Addition of Lysine to pNW1437	pNW1084
NSW2115	TTTAATGTCGTTAAATGC**TTT***TTGAAAATATAAATTTTC*		
NSW2116	*GAAAATTTATATTTTCAA***GAA**GCATTTAACGACATTAAA	Addition of glutamic acid to pNW1437	pNW1082
NSW2117	TTTAATGTCGTTAAATGC**TTC***TTGAAAATATAAATTTTC*		
NSW2218	GCTAGTCGACCGGGGAAGAGGATGAAAAAAGCAATGAAG	*sipW-tasA* amplification (SalI/SphI)	pNW1619
NSW2219	GCTAGCATGCCTAGAGCTGTTATTAATTTTTATCCTCGCTATGC		
NSW1892	CAGGGGCCCCTGGGATCC*GAAAATTTATATTTTCAA*GCATTTAACGACATTAAA	Insertion of TEV protease site to pNW543	pNW1437
NSW1893	TTTAATGTCGTTAAATGC*TTGAAAATATAAATTTTC*GGATCCCAGGGGCCCCTG		
NSW660	GCATGGATCCGCATTTAACGACATTAAATCAA	*tasA* amplification (BamHI/XhoI)	pNW543
NSW661	GCATCTCGAGTTAATTTTTATCCTCGCTATGCGA		
NSW1308	GCATGGATCCCTCTTCCCATTTGGACATGTG	Upstream *tapA* region for deletion	pNW685
NSW1332	GGTAAGATATGTTTCGATTGGTCGACATGC		
NSW1333	GCATGTCGACCAGAAGGAAAGCGGGGAAGAG	Downstream *tapA* region for deletion	
NSW1334	GCATGAATTCATATCGAAACCTGTTGCCAGG		

Primer	Sequence (5'-3')	Purpose	Plasmid
NSW2126	CGAAAATTTATATTTTCAATTTTTCAGTGATAAAGAAGTG	Removal of serine from pNW1096	pNW1099
NSW2127	CACTTCTTTATCACTGAAAAA*TTGAAAATATAAATTTTC*G		
NSW1857	GCATGTCGACATAAAAGGGGAGCTTACCATGGGTATGAA	*tasA* gene amplification (SalI/SphI)	pNW1432
NSW1858	ATGCGCATGCTTATTAATTTTTATCCTCGCTATGC		
NSW1896	ATGCGTCGACTTTTACAGGAGGTAAGATATGTTTCG	Amplification of tapA operon: *tapA-sipW-tasA*	pNW1804
NSW2219	GCTAGCATGCCTAGAGCTGTTATTAATTTTTATCCTCGCTATGC		
NSW1530	GCTCAGGATCCTAACTCACATTAATTGCG	Amplification of P_{IPTG}-*tasA-lacI*	pNW1434
NSW1865	TGCAGGGATCCGACTCTCTAGCTTGAGGC		
NSW2220	AGAGGATGAAAAAAGCAATGAAGCTG**TAATAA**AATATTTTATACGTG	Replacing 3rd and 4th sipW codons with stop codon	pNW1631
NSW2221	CACGTATAAAATATT**TTATTA**CAGCTTCATTGCTTTTTTCATCCTCT		

* Underlined indicates insertion of a restriction site, the bold the insertion of codons for specified amino acids, and highlighted in italic underlined text represents the nucleotide sequence that encodes a TEV protease cleavage site.

Table S4 Single nucleotide polymorphisms identified by genomic sequencing

Genome co-ordinates	NCIB3610 & NRS5267	NRS5248	Mutation Type	Codon Substitution [a]	Locus Tag
**490560**	C	A			
**490580**	GT	GTT			
**2552843**	A	AGAAAC ATGAAAC CGAATAC	Frame shift + stop codon	gat/gaAACATGAAACCGAATACGAt	*sinR*
**3874225**	T	C			
**4087227**	A	T	None	ggA/ggT	*yxcE*
**4087244**	G	A	Missense	aGc/aAc	*yxcE*
**4087248**	A	T	None	ggA/ggT	*yxcE*
**4087254**	A	G	None	acA/acG	*yxcE*
**4087260**	T	A	None	ggT/ggA	*yxcE*
**4087271**	T	C	Missense	aTc/aCc	*yxcE*
**4087272**	C	A	None	atC/atA	*yxcE*
**4087287**	A	C	None	ccA/ccC	*yxcE*
**4087317**	C	A	None	ggC/ggA	*yxcE*
**4087320**	C	A	None	ccC/ccA	*yxcE*
**4087332**	T	C	None	tcT/tcC	*yxcE*
**4087338**	C	A	None	ggC/ggA	*yxcE*
**4087347**	C	T	None	ccC/ccT	*yxcE*
**4087366**	A	G	Missense	Atc/Gtc	*yxcE*
**4087380**	T	C	None	atT/atC	*yxcE*
**4087491**	C	T	None	gaC/gaT	*yxcD*

a. Uppercase font indicates nucleotide difference from NCIB3610

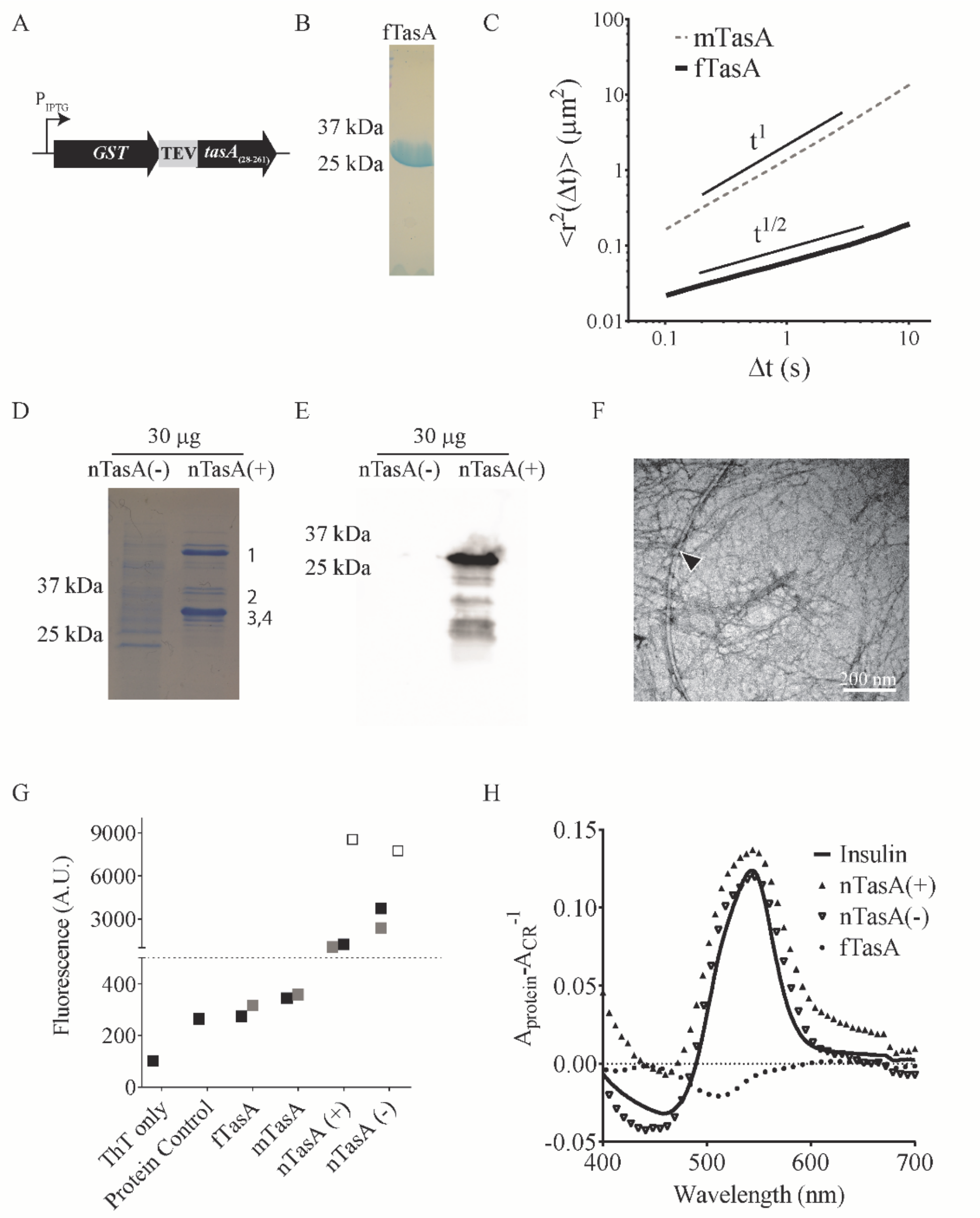

Figure S1: **Purification and characterisation of recombinant fibre-forming TasA and natively extracted nTasA.** (**A**) Schematic of expression construct used for purification of TasA(28-261) (fTasA) using the plasmid backbone pGex-6-P-1 with TEV protease cleavage site. (**B**) SDS-PAGE analysis of 10 µg purified fTasA with molecular mass of 25731 kDa as calculated by LC-MS analysis **(C)** Mean square displacement (MSD) versus lag time for 2 mg/ml fTasA (solid line) and mTasA (dashed line). The slope of the MSD for mTasA scales as t^1 indicative of a viscous fluid medium. In contrast, the MSD slope for fTasA scales as $t^{1/2}$ which is indicative of a viscoelastic medium. **(D)** SDS-PAGE analysis of 30 µg extracted nTasA(-) and nTasA(+) with mass spectrometry identification of bands labelled: 1 (AppA oligonucleotide binding protein 50% coverage); 2 (Flagellin 63% coverage); 3 (TasA 76% coverage); and 4 (Chitosanase 63% coverage) respectively. **(E)** Immunoblot analysis of 30 µg nTasA(-) and nTasA(+) extracts using anti-TasA antibody. **(F)** Transmission electron microscopy images of

nTasA(+) stained with uranyl acetate with arrow highlighting flagella. **(G)** Maximum ThT fluorescence of ThT-only, protein control, nTasA(+) and nTasA(-) extracts showing 3 independent single reads. Recombinant fTasA and mTasA are the median read from 2 independent time course experiments. The different colour boxes represent data from independent experiments. **(H)** Absorbance of Congo Red dye in presence of fTasA, nTasA(+) and nTasA(-) subtracted from background alongside insulin amyloid fibril positive control.

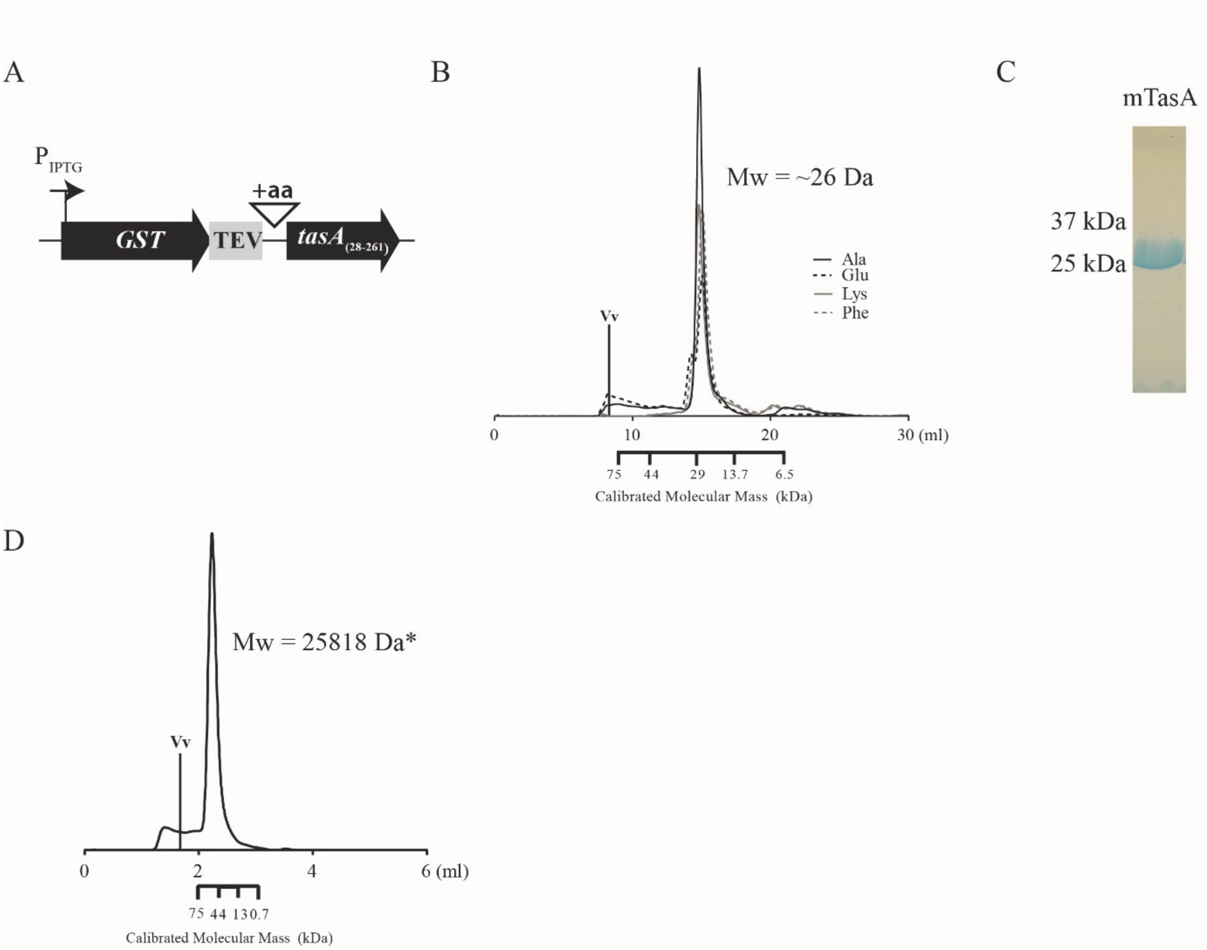

Figure S2: **Purification and characterisation of recombinant monomeric TasA (A)** Sequence schematic of amino acid insertion, indicated by 'aa'. **(B)** Size exclusion chromatography (SEC) analysis (Superdex 200 10/300 GL) of N-terminal tagged TasA where the amino acids at N-terminus indicated (Ala, Glu, Phe, Lys). **(C)** SDS-PAGE analysis of 10 µg purified serine tagged TasA (mTasA) with molecular mass of 25818 kDa as calculated by LC-MS. **(D)** SEC of mTasA (Superdex 200 5/150 GL).

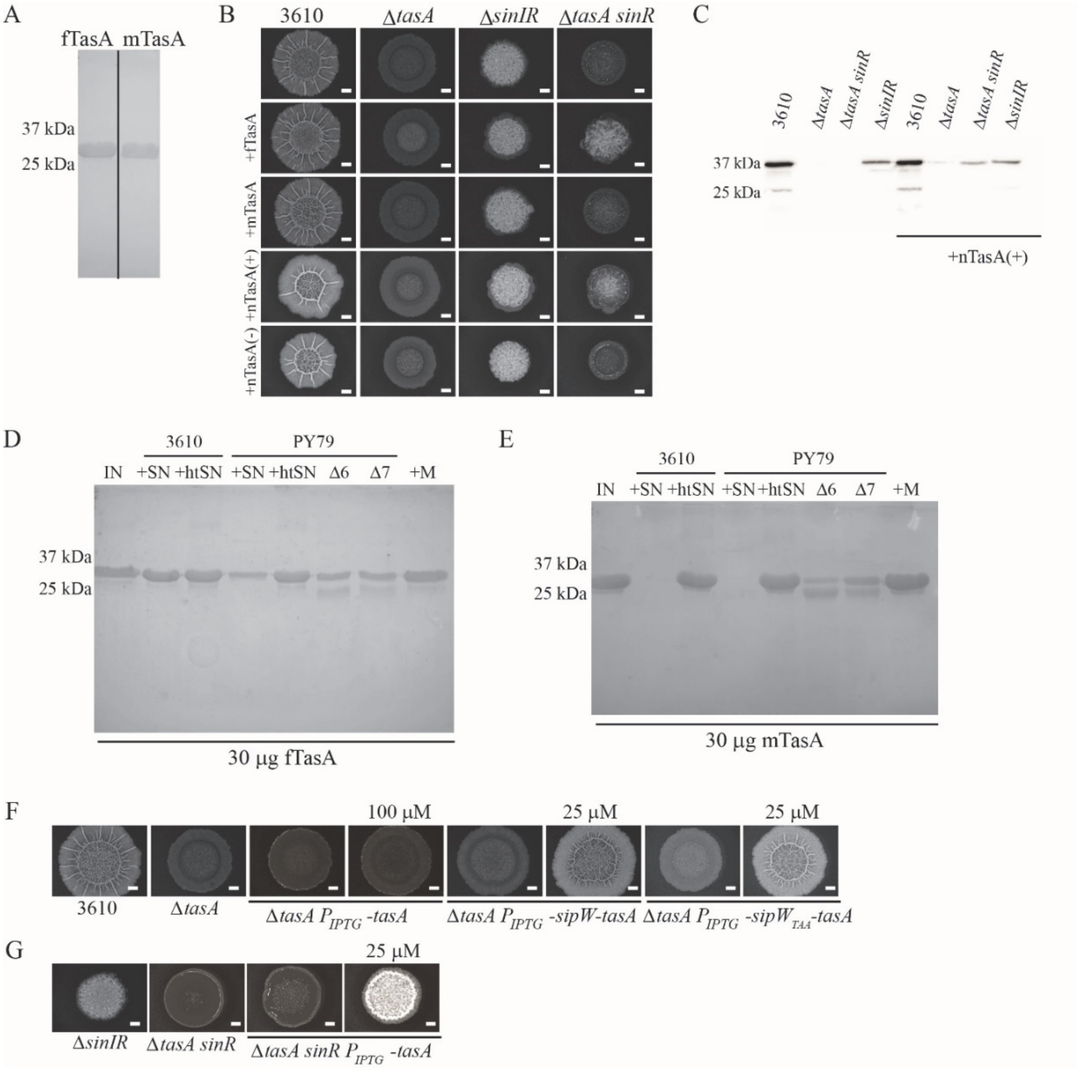

Figure S3: Recombinant fTasA is biologically active (A) Representative SDS-PAGE analysis of fTasA and mTasA, showing 10 µg protein used in (B). **(B)** Biofilm phenotypes of NCIB3610, Δ*tasA* (NRS5267) *sinIR* (NRS2012) and Δ*tasA sinR* (NRS5248) with the *ex vivo* addition of 10 µg purified protein or 30 µg native extract as indicated. Images shown in Fig. 3A, C are repeated here. **(C)** Immunoblot blot analysis of biofilm lysate collected from controls and *ex vivo* addition of nTasA(+) challenged with α-TasA antibody. **(D-E)** Integrity of 30 µg fTasA and mTasA incubated for 24 hrs at 37°C analysed by SDS-PAGE. The protein (IN) was incubated with filtered spent supernatants collected from NCIB3610 and PY79 (+SN) and the same supernatants after heat inactivation at 100°C (+htSN); supernatants from exoprotease deficient strains derived from PY79 (Δ6 and Δ7) were also used. (+M) indicates media only control. **(F-G)** Control biofilms for the genetic complementation of Δ*tasA* and Δ*tasA sinR* as shown in Fig 2D-E in absence and presence of IPTG at concentrations indicated. Whole genome sequencing of Δ*tasA sinR* (NRS5248) strain identified a duplication of region Phe65-Iso71 leading to frame shift and stop codon as highlighted in Table S4.

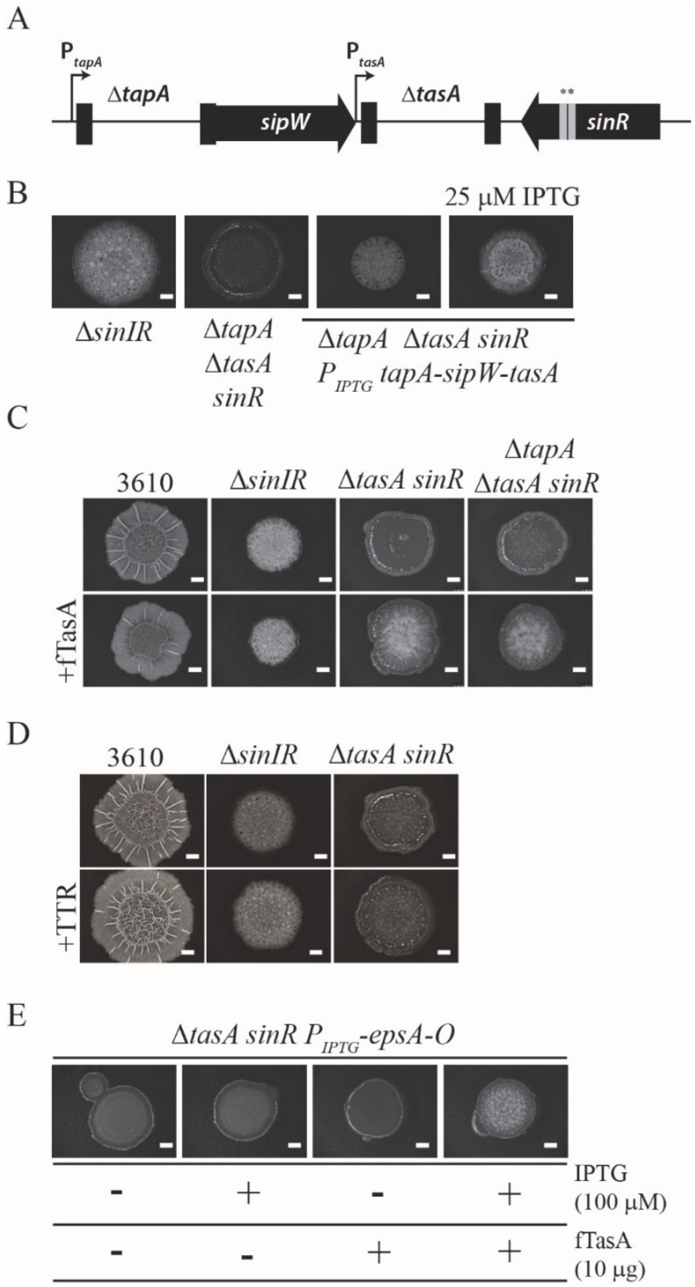

Figure S4: The biological activity of fTasA is independent of *tapA* but is dependent on specific matrix interaction. (**A)** Sequence schematic of Δ*tapA* Δ*tasA sinR* (NRS5749). **(B)** Biofilm phenotype of complementation in presence and absence of 25 µM IPTG (NRS5763). **(C)** Biofilm phenotypes of NCIB3610, Δ*tasA sinR* (NRS5248) and Δ*tapA* Δ*tasA sinR* (NRS5749) with the addition of 10 µg fTasA *ex vivo.* **(D)** Biofilm phenotype of Δ*tasA sinR* in presence and absence of the fibrous amyloid protein transthyretin (+TTR). **(E)** Biofilm phenotype of Δ*tasA sinR* P_{IPTG}-*eps* (NRS5421) in presence or absence of 100 µM IPTG and presence or absence of 10 µg fTasA where indicated by ” –“ and “+” respectively.

```
TasA_B_massi  ----------------------------------------------------------------- 000
CalY_B_cereus MSLKKKLGMGVASAALGLSLIGGGTFAFFSDKEVSNNTFAAGTLDLTLNP-----KTLVDIKDLK 060
TasA_B_cereus MSLKKKLGMGVASAALGLSLIGGGTFAFFSDKEVSNNTFAAGTLDLALNP-----KTIVDIKDLK 060
TasA_B_meth   MSIKKKLGLGIASAALGLSLVGGGTFAYFNDFAETSGTFASGTLDLNARP-----TTIINVDNIK 060
TasA_B_coah   MGIKQKLGLGVASAALGLSLVGGGTYAYFNDTETSTNTFAAGTLDLSVDP-----EVIINVDNIK 060
TasA_B_maris  MTIKKKLGLGVASAALGLSLIGGGTFAAFNDTATINNHFASGTLDLSVEKNGNNKPLNFDISNMK 065
TasA_B_selen  MSLKKKITMGALSATLGLSLVAGGTWAAFNDVETVSAGMEAGTLKLDLKK-YENKPFNFQISDLK 064
TasA_B_azto   MSIKKKLGLGVASAALGLSLIGGGTWAAFNDIETVGSSFAAGTLNLDVNN-GDGATALFDLSNLK 064
TasA_B_firm   MGIKKKIGLGVASAALGISLVGGGTWAAFNDVETLSASYAAGKLDLTAAD---TTSTGINLSNLK 062
TasA_B_pum    ----------------------------------------------------------------- 000
TasA_B_safe   MAMKKSIRLGVLSGALGLALIGGGTWAAFNDIEKANAVYSTGELDLSAKE----NSGAINLANLK 061
TasA_B_xia    ----------------------------------------------------------------- 000
TasA_B_strat  MAMKKSIRLGVLSGALGLALIGGGTWAAFNDVETANAVYSTGELNLSAKE----NSGAINLANIK 061
TasA_B_alti   MAMKKSIRLGVLSGALGLALIGGGTWAAFNDVETANAVYSTGELNLSAKE----NSGAINLANIK 061
TasA_B_lich   MGTKKKLGLGVASAALGLALVGGGTWAAFNDIETTQATYAAGTLDLNAKD----TSARVNLSNLK 061
TasA_B_sono   MGTKKKIGLGVASAALGLALVGGGTWAAFNDIETTQATYAAGTLDLNAKE----TSARVNLSNLK 061
TasA_B_sub    MGMKKKLSLGVASAALGLALVGGGTWAAFNDIKSKDATFASGTLDLSAKE----NSASVNLSNLK 061
TasA_B_amy    MGMKKKLSLGVASAALGLALVGGGTWAAFNDVKSTDATFASGTLDLSAKE----QSANVNLSNLK 061
TasA_B_sia    MGMKKKLSLGVASAALGLALVGGGTWAAFNDVKSTDATFASGTLDLSAKE----QSANVNLSNLK 061
TasA_B_atro   MGMKKKLSLGVASAALGLALVGGGTWAAFNDIKTTEATFASGTLDLSAKE----NSANVSLSNLK 061

TasA_B_massi  ----------LENNGTLDISSIDLATSYSI---------------TDAAGDN-VDDFGKHIKVLF 039
CalY_B_cereus PGDSVKKEFLLKNNGTLAIKDVKLATKYSI---------------TDAKGDNAGEDFGKHIKVKF 110
TasA_B_cereus PGDSVKKEFLLQNNGTLAIKDVKLATKYSI---------------ADAKGDNAGEDFGKHIKVKF 110
TasA_B_meth   PGDKMLREFKLINDGSLDIAKVLLRTDYTV---------------KDAQNNNGNEDFGKHIKVNF 110
TasA_B_coah   PGDWMNRTFKLNNDGSLDISRVLLTTDYSV---------------TDAGGDN-INDMGEHIRVNF 109
TasA_B_maris  PGDSVQREFKLRNAGTLAIKDILLTVQAGGYS--------------NNESGATIDEFLSQFEVTL 116
TasA_B_selen  PGDKMTRNIKLVNSGTLAIRDVLMSIESVQFADYVPAEGEAGYEDDDTWGDNNVVQYLNQFRVTV 129
TasA_B_azto   PGDTMEREFLLSNAGSLAIKDVLMTTAITSFTQGE-----NEYVNIHGATDNSQEDFLNQFNVNI 124
TasA_B_firm   PGDVFQKEFELDNVGTLAIKDVLLKLTHSNFVDYV-----GGD-DTDIWGKNSAADFLRQFTITV 121
TasA_B_pum    ---------HFDNKGSLAINQVLMSLDYSQFT--------DGS-SAKNGGENTAEEFLSQFQVSV 047
TasA_B_safe   PGDRIKKEFHFDNKGSLAINQVLMSLDYSQFT--------DGS-SAKNGGKNTAEEFLSQFQVSV 117
TasA_B_xia    ---------NFENKGSLAINQVLMSLDYSQFK--------DGT-SAKNGGKNTAEEFLSQFQVSV 047
TasA_B_strat  PGDRIKKEFNFENKGSLAINQVLMSLDYSQFQ--------DGT-SAKNGGKNTAEEFLSQFQVSV 117
TasA_B_alti   PGDRIKKEFNFENKGSLAINQVLMSLDYSKFQ--------DGT-SAKNGGKNTAEEFLSQFQVSV 117
TasA_B_lich   PGDKFTKDFEFKNDGSLAIKEVLMQVGYSNFV--------DGN--AKNGGKSTAEDFLKQFKVSV 116
TasA_B_sono   PGDKFKKEFTFKNDGSLAIKEVLMQMGFSNFV--------DAN--AKNGGKNSAEDFLKQFKVSV 116
TasA_B_sub    PGDKLTKDFQFENNGSLAIKEVLMALNYGDFK--------A-----NGGSNTSPEDFLSQFEVTL 114
TasA_B_amy    PGDKLTKDFEFRNNGSLAIKEVLMALNFTDFK--------G-----AKKGNESAEDFLSQFEITV 114
TasA_B_sia    PGDKLTKDFEFRNNGSLAIKEVLMALNFTDFK--------G-----AKKGNESAEDFLSQFEITV 114
TasA_B_atro   PGDKLTKDFEFRNNGTLAIKEVLMALNYSGFN--------T-----AKGDNDSPEDFLSQFQITL 114

TasA_B_massi  LENADKTGDGWIIGDYNDIISE---TTLYDLQNMTPDAVE------------------------- 076
CalY_B_cereus LWNWDK---------QSEPVYE---TTLADLQNVDPDVLA------------------------- 138
TasA_B_cereus LWNWDK---------QSEPVYE---TTLADLQNVDPDVLA------------------------- 138
TasA_B_meth   LYNADK---------TDNVIYQ---TTLDQLKSMTPDAIE------------------------- 138
TasA_B_coah   LQNIDKSGI----VKPNNIIYS---TTLHDLKGTAPDAVQ------------------------- 142
TasA_B_maris  FKVNQSNDEY---DDFNSLVKTGQVLTLNDLVKGT---------------------L-----SEK 152
TasA_B_selen  MQVGAEGGSG---GFPRELIPADKDVRLGDFYLASGSLAGDDSKLNDGVTQADINTALSNVWGAV 191
TasA_B_azto   IDVDRQIT-----------VVQN--KTLKDLLTE------------------------------- 145
TasA_B_firm   ISSGTEGGN----GYSKDIVK-D--VNLFDFIEMTAGKGV---------------PP--NA-GSV 161
TasA_B_pum    LTVGAEGGN----GYPKNIILDH--ANLLDLHQLTSKQDQ---------------TAFEKL-RHA 090
TasA_B_safe   LTVGAEGGN----GYPKNIILDH--ANLLDLHQLTSKQDQ---------------TAFEKL-RHA 160
TasA_B_xia    LTVGAEGGN----GYPKNIILDH--ANLLDLHLLTSKQDQ---------------TAFE---RHA 088
TasA_B_strat  LTVGAEGGN----GYPKNIILDH--ANLLDLHLLTSKQDQ---------------TAFEKL-RHA 160
TasA_B_alti   LTVGAEGGN----GYPKNIILDH--ANLLDLHLLTSKQDQ---------------TAFEKL-RHA 160
TasA_B_lich   LTVGVEGGN----GYPKNIILDE--ANLYDLYNMSAKKDK---------------SAYEKV-KKA 159
TasA_B_sono   LTVGVEGGN----GYPKNIILDS--ANLYDLYNMSAKKDK---------------SSMEKI-KKL 159
TasA_B_sub    LTVGKEGGN----GYPKNIILDD--ANLKDLYLMSAKNDA---------------AAAEKI-KKQ 156
TasA_B_amy    LTVGKEGGN----GYPKNIILKA--ASLKDLYLMSTKQDK---------------AAAEAI-SKH 156
TasA_B_sia    LTVGKEGGN----GYPKNIILKA--ASLKDLYLMSTKQDK---------------AAAEAI-SKH 156
TasA_B_atro   LTVGKEGGN----GYPKNIILKA--ANLKDLYLMSAKQDK---------------TAAEKI-GQL 156

TasA_B_massi  ---------NLNSFLTWLLGLGGEDSGLPAGTSD--EMYVAFEFVDN-------GQDQNEFQGDS 123
CalY_B_cereus ---------KDI----FAPEWAE-NGGLAPNSED--YLWVQFVFEDD-------GKDQNKFQGDS 180
TasA_B_cereus ---------KDI----FAPEWGE-KGGLEAGTED--YLWVQFVFEDD-------GTDQNKFQGDS 180
TasA_B_meth   ---------NEV----FNKWLDEKGGKLAAGTSD--TLYVQFEFVDN-------GQDQNIFQGDS 181
TasA_B_coah   ---------EKI----WSFFGENS--GLKTGTSD--NFYVQFEFVDN-------QDDQNEFQGDS 183
TasA_B_maris  IKTDY-MSEGKINLAPIGIDNNP--KGLT--KTSANSVAMIVKFKEDDRKDSEGKYLQNKFMNNK 212
TasA_B_selen  DHTYIDAASRRINGATIN---PNEWTGLPVNPNDDDVVEISIEFVKDNTLNDKGTYVQNKYQGDT 252
TasA_B_azto   ----------TPNLAPI-NTNDPRYSGIPLNPADTENIRIQITFKEDPTVGINGEQDQNKYQGDS 199
TasA_B_firm   DSNYYDAESGRINVITKTGA-DQSYHGLPVNPDDKDKVLFTIKFEDIDDKDNKGRQIQNKYQGDS 225
TasA_B_pum    VDEKFLHESGKINVATVDGTVAPEYDGIPKNPFDYDKVEMIIEFINDQTKDKAGHYIQNKYQGDA 155
TasA_B_safe   VDEKFLHESGKINVATVDGTVAPEYDGIPKNPFDYDKVEMIIEFINDQTKDKAGHYIQNKYQGDA 225
TasA_B_xia    VDEKFLHESGKINVATVDGTAAPEYDGIPKNPFDYDKMEMIIEFVNDQTKDKAGHYIQNKYQGDA 153
TasA_B_strat  VDEKFLHESGKINVATVDGSTAPEYDGIPKNPFDYDKMEMIIEFVNDQTKDKAGHYIQNKYQGDA 225
TasA_B_alti   VDEKFLHESGKINVATVDGSTAPEYDGIPKNPFDYDKMEMIIEFVNDQTKDKAGNYIQNKYQGDA 225
TasA_B_lich   IEPAFLHDNGKINVATIDGKTAPEYDGIPKDPYDFDKVQLVIEFVNDKTTDASGRMVQNKYQGDS 224
TasA_B_sono   IEPGFLHDNGKINVATIKGKTAPQYDGIPKDPYDFDKVEMVIEFINDTTTDSNGNMIQNKYQGDS 224
TasA_B_sub    IDPKFLNASGKVNVATIDGKTAPEYDGVPKTPTDFDQVQMEIQFKDDKTKDEKGLMVQNKYQGNS 221
TasA_B_amy    IDPKFLSESGKVNVATINGKTAPEYDGVPKTPADYDQVRMEIKFKNDTAKTADGLSVQNKFQGNA 221
TasA_B_sia    IDPKFLSTSGKVNVATINGKTAPEYDGVPKTPVDYDQVRMEIQFKNDTAKGADGLSVQNKFQGNA 221
TasA_B_atro   IDSKFLSESGKINVATINGKTAPEYDGVPKTPVDYDQVQMVIEFKNDNAKDANGQMVQNKYQGNS 221

TasA_B_massi  LELEWTFTAHQTDGESK------------------------------ 140
CalY_B_cereus LNLEWTFNASQTDGEEK------------------------------ 197
TasA_B_cereus LNLEWTFNASQTDGEEK------------------------------ 197
TasA_B_meth   LELKWTFEGKQGAGQYK------------------------------ 198
TasA_B_coah   LSLEWTFEAKQTDGERR------------------------------ 200
TasA_B_maris  VDFKFNLEATQWNGVKIDTNDA---------NGEINNGNQVA----- 245
TasA_B_selen  ATVNFQFEARQWGGQEVTDSDIGAGLKGADKEGYIQTNERANNGN-- 298
TasA_B_azto   AQISFTLEATQWDGLTTD---------QTKDNGYLDINKEANSN--- 234
TasA_B_firm   ITLDFNFEARQWGGLTIE-------ESHVDEDGYISENEKSHSED-- 263
TasA_B_pum    IQIDLSFEATQWNGLTINP------KKHTDEKGYVKENEKAHSEDKK 196
TasA_B_safe   IQIDLSFEATQWNGLTINP------KKHTDEKGYVKENEKAHSEDKK 266
TasA_B_xia    IQIDLSFEATQWNGLTINP------KEHTDEKGYVKENEKAHSEDKK 194
TasA_B_strat  IQVDLSFEATQWNGLTINP------KKHTDEKGYVKENEKAHSEDKK 266
TasA_B_alti   IQVDLSFEATQWNGLTINP------KKHTDEKGYVKENEKAHSEDKK 266
TasA_B_lich   VQLDFSFEATQWNGLTIDG------KKHADEKGYVKENERAHSEDK- 264
TasA_B_sono   VQLDFSFEGTQWNGLTIDG------NKHADEKGYVKENERAHSEDK- 264
TasA_B_sub    IKLQFSFEATQWNGLTIK-------KDHTDKDGYVKENEKAHSEDKN 261
TasA_B_amy    ISLQFSFEATQWNGLTIT-------KDHTDKDGYVKENEKAHSEDKN 261
TasA_B_sia    ISLQFSFEATQWNGLTIT-------KDHTDKDGYVKENEKAHSEDKN 261
TasA_B_atro   IQLNFSFEATQWNGLTIK-------KDHTDKNGYIKENEKAHSEDK- 260
```

Figure S5: Alignment. Alignment of 20 TasA orthologues pulled from BlastP (Altschul *et al.*, 1990; Altschul *et al.*, 1997) of *B. subtilis* TasA sequence (*Bacillus azotoformans* (B_azto) TapA (WP_003329523), TasA (WP_035196521.1). *Bacillus firmus* (B_firm) TapA (WP_082139007), TasA (WP_035326610.1). *Bacillus selenatarsenatis* (B_selen) TapA (WP_084135527), TasA (WP_041967097.1). *Bacillus licheniformis* (B_lich) TapA (WP_075747486), TasA (WP_043927876.1). *Bacillus sonorensis* (B_sono) TapA (WP_006637531), TasA (WP_006637529.1). *Bacillus marisflavi* (B_maris) TapA (WP_082139781), TasA (WP_048012492.1). *Bacillus amyloliquefaciens* (B_amy) TapA (WP_063094776), TasA (WP_044802563.1). *Bacillus siamensis* (B_sia) TapA (WP_029575370), TasA (WP_045926714.1). *Bacillus atrophaeus* (B_atro) TapA (WP_061670003), TasA (WP_010789194.1). *Bacillus stratosphericus* (B_strat) TapA (WP_039964022), TasA WP (007501219.1). *Bacillus altitudinis* (B_alti) TapA (WP_073413951), TasA (WP_039166017.1). *Bacillus xiamenensis* (B_xia) TapA (WP_034739525), TasA (WP_008360245.1). *Bacillus pumilis* (B_pum) TapA (WP_041106857), TasA (WP_041106853.1). *Bacillus safensis* (B_safe) TapA (WP_075623495), TasA (WP_034280222.1). *Bacillus methanolicus* (B_meth) TapA (None), TasA (WP_004434174.1). *Bacillus massiliosenegalensis* (B_massi) TapA (None), TasA (WP_019154446.1). *Bacillus coahuilensis* (B_coah) TapA (None), TasA (WP_010174983.1). *Bacillus cereus* (B_cer) TapA (None), TasA (WP_002201283.1)) by Clustal Omega (Sievers *et al.*, 2011). Signal sequence as predicted by SignalP v4.2 (Petersen *et al.*, 2011) underlined, 100% sequence identity highlighted in black.

A

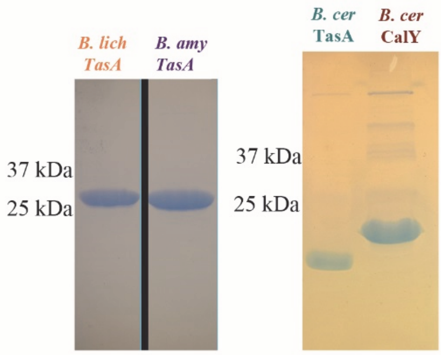

B

B. amyloliquefaciens Protein sequence coverage: 100%
Matched peptides shown in ***bold red***.

**1**	**AAFNDVKSTD**	**ATFASGTLDL**	**SAKEQSANVN**	**LSNLKPGDKL**	**TKDFEFRNNG**
**51**	**SLAIKEVLMA**	**LNFTDFKGAK**	**KGNESAEDFL**	**SQFEITVLTV**	**GKEGGNGYPK**
**101**	**NIILKAASLK**	**DLYLMSTKQD**	**KAAAEAISKH**	**IDPKFLSESG**	**KVNVATINGK**
**151**	**TAPEYDGVPK**	**TPADYDQVRM**	**EIQFKNETAK**	**TADGLSVQNK**	**FQGNAISLQF**
**201**	**SFEATQWNGL**	**TITKDHTDKD**	**GYVKENEKAH**	**SEDKN**	

B. licheniformis Protein sequence coverage: 96%
Matched peptides shown in ***bold red***.

**1**	**AFNDIETTQA**	**TYAAGTLDLN**	**AKDTSARVNL**	**SNLKPGDKFT**	**KDFEFKNDGS**
**51**	**LAIKEVLMQV**	**GYSNFVDGNA**	**KNGGKSTAED**	**FLKQFKVSVL**	**TVGVEGGNGY**
**101**	**PKNIILDEAN**	**LYDLYNMSAK**	**KDKNAYEK**VK	**KAIEPEFLHD**	**NGKINVATIN**
**151**	**GKTAPEYDGI**	**PKDPYDFDKV**	**QLVIEFVNDK**	**TTDASGRMVQ**	**NKYQGDSVQL**
**201**	**DFSFEATQWN**	**GLTIDGKKHA**	**DEKGYVKENE**	**R**AHSEDK	

B. cereus TasA Protein sequence coverage: 100%
Matched peptides shown in ***bold red***.

**1**	**FFSDKEVSNN**	**TFAAGTLDLT**	**LNPKTLVDIK**	**DLKPGDSVKK**	**EFLLQNSGSL**
**51**	**TIKDVKLATK**	**YTVKDAKGDN**	**AGEDFGKHVK**	**VKFLWNWDKQ**	**SEPVYETTLA**
**101**	**DLQKVDPDLL**	**AKDIFAPEWG**	**EKGGLAAGTE**	**DYLWVQFEFV**	**DDGKDQNIFQ**
**151**	**GDTLNLEWTF**	**NANQEAGEEK**			

B. cereus CalY Protein sequence coverage: 97%
Matched peptides shown in ***bold red***.

**1**	**FFSDKEVSNN**	**TFAAGTLDLE**	**LNPSTVVNVS**	**NLKPGDTIEK**	**EFKLENKGSL**
**51**	**DIKKVLLKTD**	**YNVEDVKKDN**	**KDDFGKHIKV**	**TFLKNVDKHE**	**TIVKQTTLDK**
**101**	**LKGDTLTAVD**	**NDLSAWFWDE**	**KGISAGK**SDK	FK**VKFEFVDN**	**GKDQNQFQGD**
**151**	**KLQLNWTFDA**	**QQTAGEEK**			

C

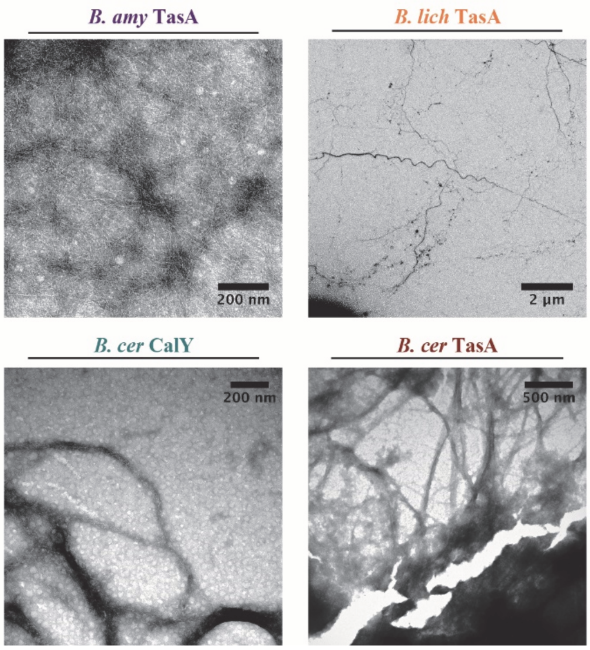

Figure S6: Characterisation of recombinant orthologous TasA. (A) SDS-PAGE of recombinant 10 µg *B. licheniformis*, *B. amyloliquefaciens* and *B. cereus* TasA and CalY generated in *E. coli*. **(B)** Coverage map of orthologue recombinant proteins sequences as determined by tandem mass spectrometry. Amino acid designated 1 signifies the first amino acid of the mature protein: *B. amyloliquefaciens* amino acid 27, *B. licheniformis* amino acid 28 and *B. cereus* for both CalY and TasA amino acid 30. **(C)** Transmission electron microscopy images of recombinant orthologue TasA stained with uranyl acetate show presence of fibres which are several micrometres long and approximately 22 nm wide, with *B. cereus* TasA fibres being significantly wider at approximately 60 nm.

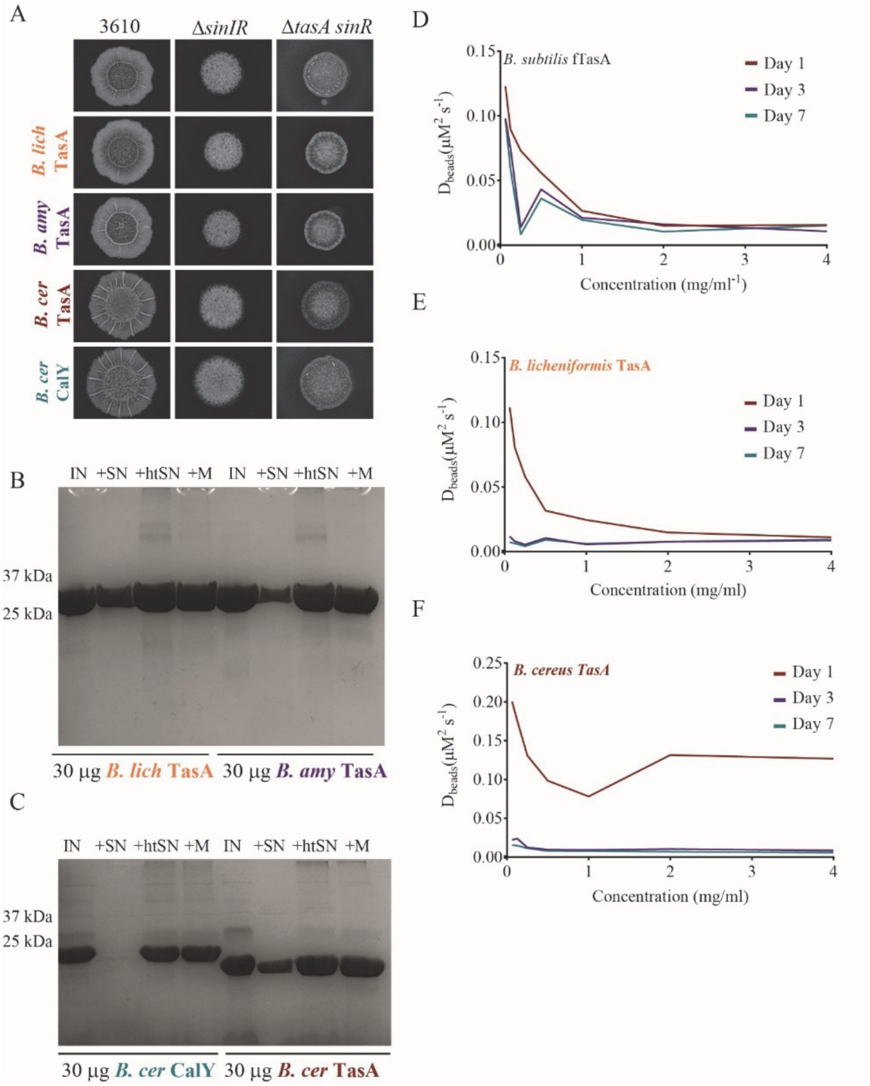

Figure S7: Complementation of Δ*tasA sinR* by recombinant orthologous fTasA. (A) Biofilm phenotypes of wild type (NCIB3610), Δ*tasA* (NRS5267), Δ*sinIR* (NRS2012) and Δ*tasA sinR* (NRS5248) strains with *ex vivo* addition of 10 µg recombinant orthologous TasA as indicated. **(B-C)** Integrity of 30 µg *B. licheniformis* TasA, *B. amyloliquefaciens* TasA and *B. cereus* CalY and TasA incubated for 24 hrs at 37°C then analysed by SDS-PAGE. The protein (IN) was incubated with filtered supernatant collected from NCIB3610 (+SN) and the supernatant after heat inactivation at 100°C (+htSN) alongside a media only control (+M). **(D-F)** Serial dilutions (from 4 mg/ml down to 0.0625 mg/ml) were prepared for *B. subtilis*, *B. licheniformis*, and *B. cereus* fTasA. Diffusion coefficients of 1µm beads were extracted from mean squared displacement curves. The diffusion coefficients were measured over successive days. The *B. licheniformis* and *B. cereus* fTasA samples gelled at lower concentrations compared to *B. subtilis* fTasA. Moreover, after 3 days, the *B. licheniformis* and *B. cereus* fTasA samples became gels for all concentrations studied indicating a slow, dynamical gelling process.
